# Virulence factors of bovine mastitis pathogens: distribution, pathogenesis, and emerging vaccines targeting virulence factors: a literature review

**DOI:** 10.3389/fvets.2025.1745390

**Published:** 2026-01-28

**Authors:** Hao Li, Ziyan Wang, Herman W. Barkema, Xiaohan Li, Deyuan Song, Meiyi Ren, Jingdi Tong, Mingchao Liu, Jian Gao, Jia Cheng

**Affiliations:** 1 Department of Clinical Veterinary Medicine, College of Veterinary Medicine, Hebei Agricultural University, Baoding, Hebei, China; 2 Faculty of Veterinary Medicine, University of Calgary, Calgary, AB, Canada; 3 Department of Clinical Veterinary Medicine, College of Veterinary Medicine, China Agricultural University, Beijing, China

**Keywords:** bacteria, bovine mastitis, pathogenicity, vaccine, virulence factors

## Abstract

Bovine mastitis, mainly caused by contagious pathogens like *Staphylococcus aureus*, *Streptococcus agalactiae*, and *Mycoplasma bovis*, environmental pathogens such as *Escherichia coli*, *Streptococcus uberis*, *Klebsiella pneumoniae*, and a more opportunistic pathogen like *Streptococcus dysgalactiae*, severely threatens dairy production. These mastitis pathogens rely on their respective virulence characteristics to exert different inflammation of the mammary gland. Meanwhile, antimicrobials remain the primary treatment for bovine mastitis, but growing resistance often causes failure. Therapeutic approaches targeting the virulence factors utilized by these mastitis-causing pathogens are expected to become effective alternatives to antimicrobial therapy in dairy farming. Therefore, the objective of this review is to investigate the prevalence and pathogenic roles of virulence genes in mastitis pathogens, with an extensive exploration of the emerging vaccination approaches targeting the virulence factors for safeguarding dairy animal health.

## Introduction

**Figure 1:**
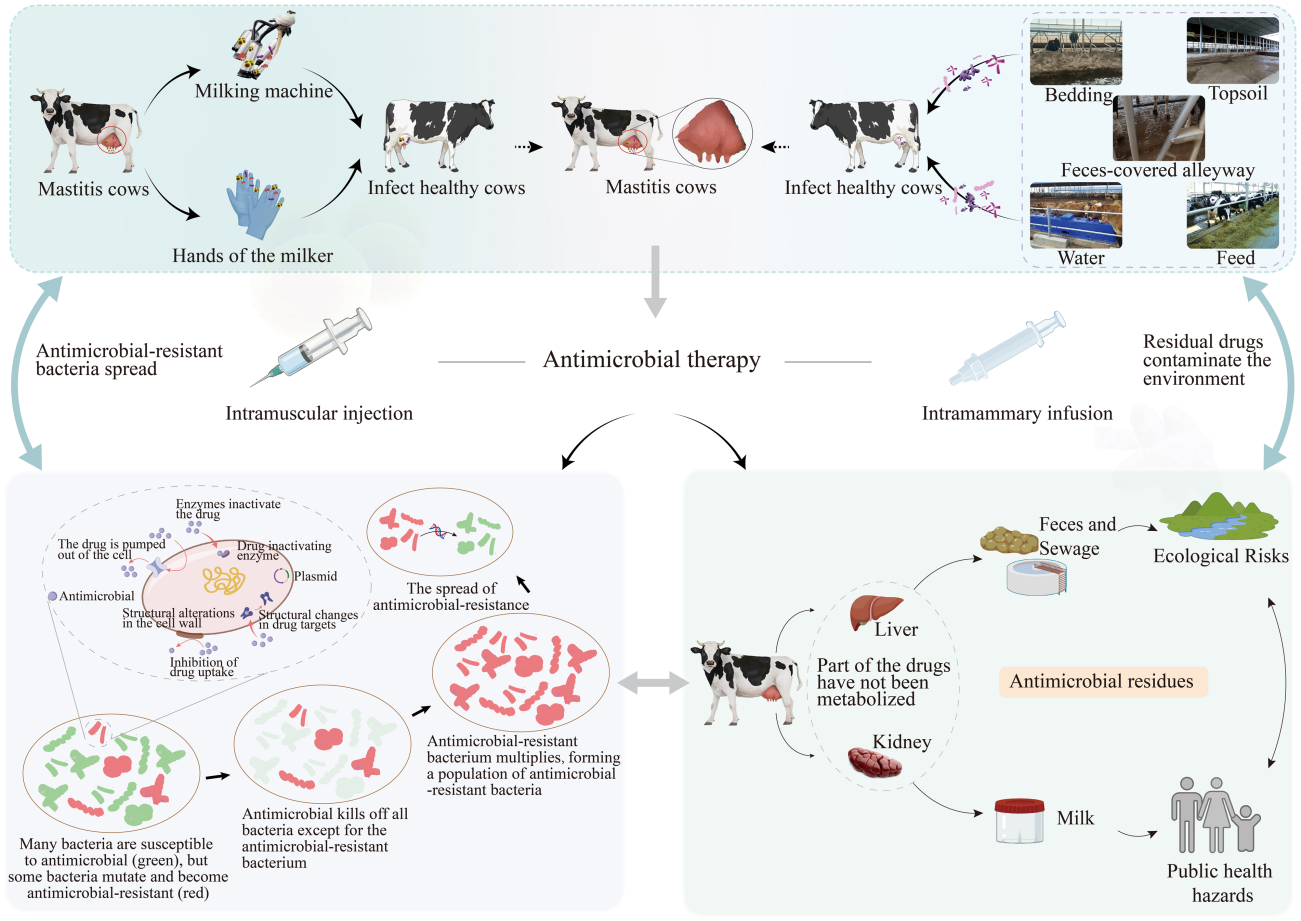
Infectious and environmental pathogens infect healthy cows, which are then treated with antimicrobials. The continuous antimicrobial pressure leads to the emergence of resistant bacterial populations and drug residues. These resistant bacteria and residual drugs then affect cows and the farm environment through different pathways. Created with BioGDP.com; reproduced with permission.

Bovine mastitis, an inflammatory disease of the mammary gland most often caused by pathogenic bacterial infection, is one of the most economically devastating diseases in dairy production due to reduced milk yield, premature culling of cows, and increased treatment costs (1), with annual losses exceeding billions of dollars (2). The pathogens causing mastitis are categorized into contagious bacteria (e.g., *Staphylococcus aureus*, *Streptococcus agalactiae*, *Mycoplasma bovis*), environmental bacteria (e.g., *Escherichia coli*, *Streptococcus uberis*, *Klebsiella pneumoniae*) and *Streptococcus dysgalactiae* which is a more opportunistic udder pathogen (3–8). Contagious pathogens are primarily transmitted between cows via milking equipment (9), which then enter the cows’ teat and udder and cause intramammary infections (IMIs). In contrast, environmental pathogens thrive in bedding, water, or soil (1, 2) (Figure 1), and these environmental pathogens can enter the mammary gland and cause disease when the cow’s teat skin is damaged or when its immune system is relatively weakened (2).

**Figure 2:**
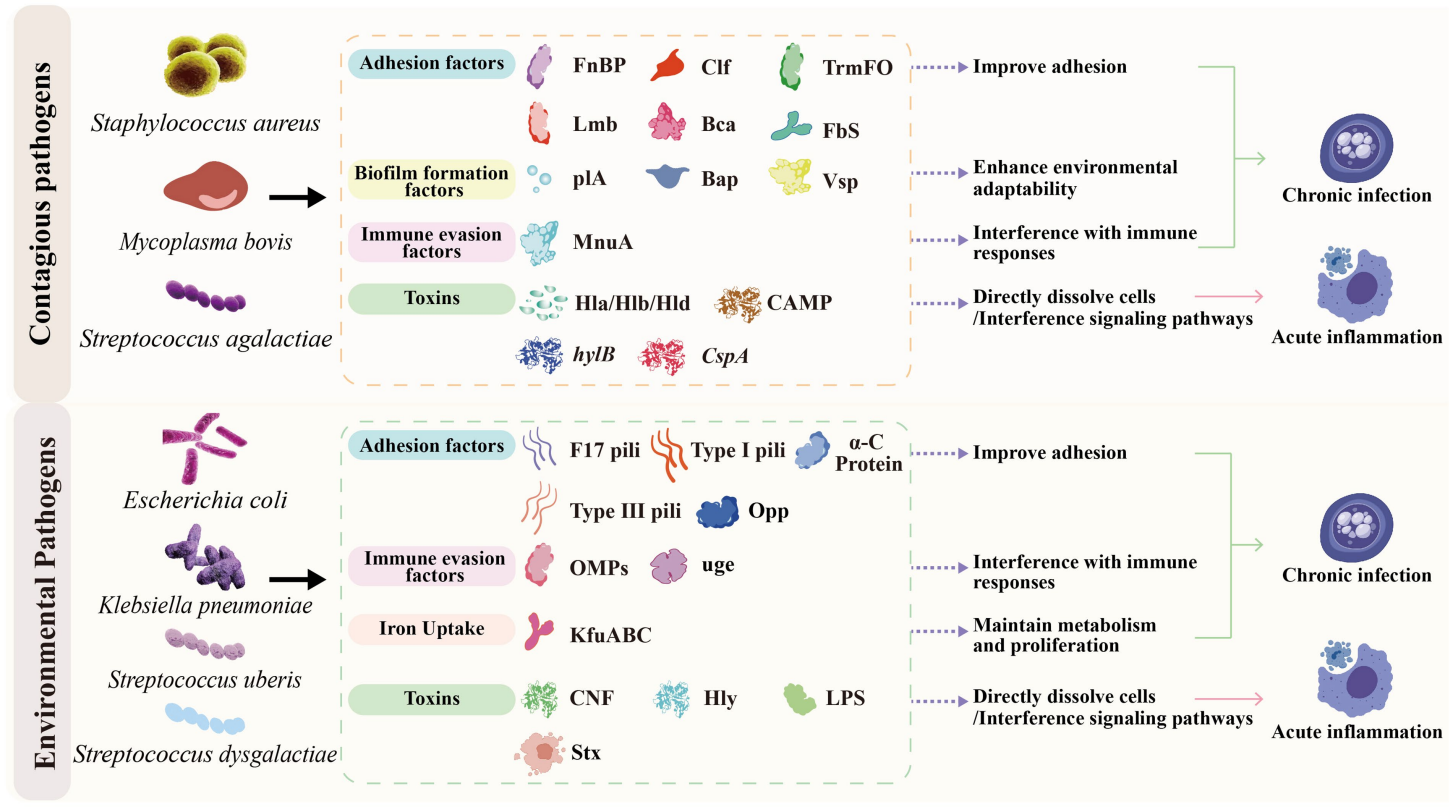
Contagious pathogens and environmental pathogens mediating chronic infections and acute inflammation through their respective virulence factors. Created with BioGDP.com; reproduced with permission.

Susceptibility to mastitis in dairy cows varies based on factors such as the cow’s age, the lactation stage, and parity (10, 11). The pathogenicity of a mastitis pathogen is due to the presence of an arsenal of virulence factors (VFs) (12–14), which are primarily proteins encoded by specific bacterial genes (15), and typically exhibit activities such as adhesion, invasion, iron acquisition, immune evasion, and toxin production (16) (Figure 2). Upon entering the mammary gland, pathogenic bacteria adhere to host cell surfaces through adhesion-related VFs, subsequently employing their array of VFs to establish colonization and exert pathogenic effects (12). For instance, *Staphylococcus aureus* and *Streptococcus agalactiae* mainly exert pathogenic effects through toxic substances such as exotoxins and surface proteins with adhesion and invasion functions (17, 18), and evade immunity through enzymatic substances (19). Additionally, *Escherichia coli* and *Klebsiella pneumoniae* primarily adhere via fimbriae, maintain survival through iron acquisition, and trigger innate immune responses in the mammary gland through toxic substances including lipopolysaccharide (LPS) (11, 20, 21).

Most cows with non-severe clinical mastitis (CM) are treated or spontaneously recover within 1 week of the onset of CM. However, cows with severe CM are usually treated with antimicrobials (22, 23). Unfortunately, pathogenic bacteria have, particularly in low- and middle-income countries, become frequently multi-resistant due to extensive use and the misuse of antimicrobials (24, 25). Therefore, there is a need to identify alternative and safe approaches to control IMIs caused by pathogens. Several interventions targeting these pathogens have emerged. Research on vaccine development and other therapeutic strategies has provided references for treating mastitis in dairy cows (26, 27). Among these, VFs are potential targets for novel therapeutic approaches (28).

Understanding the distribution patterns and pathogenic mechanisms of these VFs is crucial for guiding treatment. However, the functional differences of VFs among pathogens remain underexplored in therapeutic design, and there is a lack of systematic integration of the distribution patterns of VFs, pathogenic mechanisms, and treatment methods. Therefore, this review aims to systematically summarize the global distribution characteristics of key virulence factors (VFs) in seven major bovine mastitis pathogens, the mechanistic roles of these VFs in intramammary infections (IMIs), and emerging therapeutic strategies targeting these VFs (particularly vaccines). The objective of this integrative research approach is to provide a valuable reference for elucidating pathogenic mechanisms and developing targeted interventions. Ultimately, it aims to promote the sustainable control of mastitis by reducing reliance on the empirical use of antibiotics.

## Methods for selection of literature

This review was conducted by searching the PubMed and Web of Science databases. Studies published between 2014 and 2025 were included, focusing on virulence genes, prevalence, pathogenic mechanisms, or vaccines of bovine mastitis pathogens; language restricted to English. Non-peer-reviewed articles, editorials, conference abstracts, studies involving non-bovine species, as well as those with incomplete data or unavailable full texts were not included. All identified records underwent initial screening based on titles and abstracts, followed by a comprehensive full-text review.

A total of 7 major pathogens were included in the final analysis. Data on gene prevalence were extracted from each study, and statistical analysis was performed using chi-square test in SPSS 26.0 (IBM Corp., Armonk, NY, USA), with a significance level set at *p* < 0.05. The detailed search strategy is provided in Supplementary material 3.

## Prevalence of virulence genes and pathogenic mechanisms of virulence factors in bovine mastitis pathogens

### Staphylococcus aureus

#### Prevalence of virulence genes in *Staphylococcus aureus*

**Figure 3:**
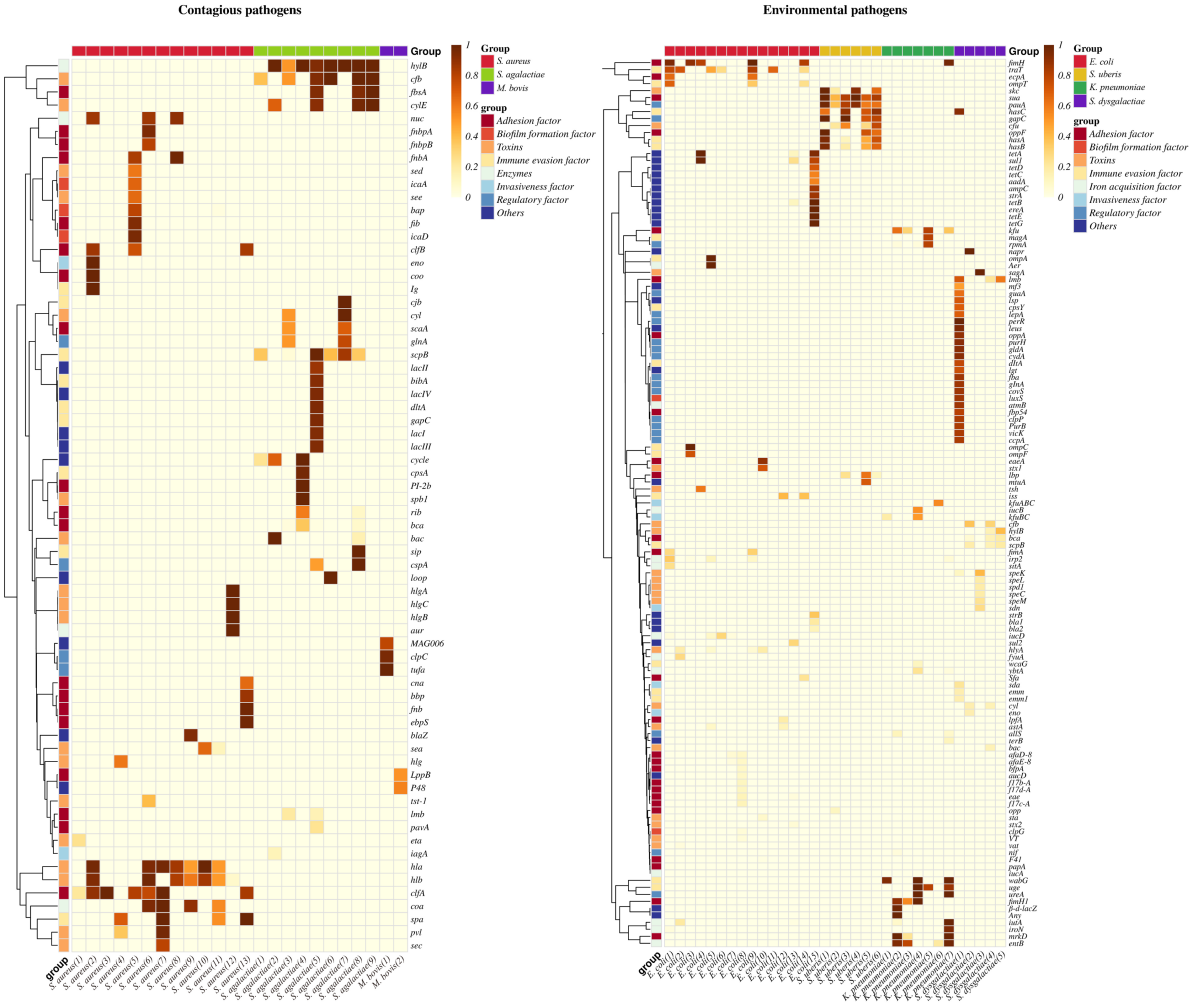
Heatmap of the prevalence of virulence genes in contagious and environmental pathogens. *S. aureus*: (1): (35), (2): (33), (3): (32), (4): (41), (5): (31), (6): (34), (7): (29), (8): (36), (9): (38), (10): (37), (11): (39), (12): (40),(13): (30). *S. agalactiae*: (1): (68), (2): (72), (3): (65), (4): (73), (5): (66), (6): (69), (7): (67), (8): (18), (9): (70). *M. bovis*: (1): (80), (2): (81). *E. coli*: (1): (94), (2): (93), (3): (95), (4): (97), (5): (90), (6): (99), (7): (100), (8): (176), (9): (91), (10): (92), (11): (98), (12): (177), (13): (178), (14): (96), (15): (101). *S. uberis*: (1): (117), (2): (118), (3): (119), (4): (120), (5): (121), (6): (122). *K. pneumoniae*: (1): (179), (2): (6), (3): (133), (4): (137), (5): (136), (6): (135), (7): (134). *S. dysgalactiae*: (1): (150), (2): (147), (3): (151), (4): (148), (5): (149).

A total of 34 virulence genes from 990 *S. aureus* isolates collected from eight countries (China, Brazil, Ethiopia, Thailand, India, Iran, South Africa, and the United States) were identified and analyzed across eight studies (Figure 3). Among them, the adhesion-related genes *clfA* and *clfB* were analyzed in 585 isolates, of which 207 were from subclinical mastitis (SCM), 373 were from mixed mastitis (herds with both CM and SCM), and 5 were from CM cases. The *clfA* gene had a high (> 70%) prevalence in Thailand (100%) (29), Iran (84%) (30), Brazil (81%) (31), China (77, 89, and 97%) (32–34), but a lower prevalence in Ethiopia at only 22% (*p* < 0.05) (35). The *clfB* gene was detected in isolates from China, Iran, and Brazil, with a prevalence of 86 and 97% in China (*p* = 0.006) (33), 84% in Iran (30), and 74% in Brazil (*p* = 0.15 for 74% vs. 84%) (31).

The prevalence of the hemolysin genes *hla* and *hlb* showed similar variation among 450 isolates from six countries, including 120 from SCM, 192 from CM, and 138 from bulk milk. Specifically, the prevalence of the *hla* gene in isolates from China was consistently high, reported at 85% (36), 94% (34), and 96% (33). Similarly, it was 96% in Thailand and 100% in Argentina (29, 37). In contrast, a lower prevalence was observed in India (49%) and South Africa (50%) (*p* = 0.001 for 50% vs. 85%) (38, 39). A parallel trend was observed for *hlb*, with higher prevalence reported in China (82–97%) (33, 34, 36), and Argentina (85%) (37). These rates differed significantly from those in India (60%) (38), South Africa (50%) (39), and the U. S. (9%) (*p* < 0.001 for 9% vs. 50%) (40). The notably low prevalence in the United States may be related to the fact that the 138 isolates were obtained from bulk tank milk.

The enzyme-encoding genes *nuc* and *coa* were examined in a total of 144 and 194 isolates, respectively. Or these, 120 isolates (58 for *nuc* and 92 for *coa*) were from SCM, 99 isolates (44 for *nuc* and 60 for *coa*) from CM, and 42 isolates (42 for both *nuc* and *coa*) from mixed SCM and CM samples. The prevalence of the *nuc* gene was consistently high: 86, 86, and 90% in China (33, 34, 36), and 100% in India (41). Similarly, the prevalence of the *coa* gene was high in Thailand (100%) (29), China (94.3%) (34), and India (89.1 and 100%) (38, 41), but a lower in prevalence was observed in South Africa, at only 55% (*p* < 0.001 for 55% vs. 89%) (39).

Similarly, the prevalence of the surface protein gene *spa* varied significantly. A total of 179 isolates were surveyed from SCM (*n* = 62) and herds with both CM and SCM (*n* = 117). The prevalence of *spa* was 100% in Thailand (29), 98.7% in Iran (30), and 93% in India (41), but it was lower in South Africa, at 53% (*p* < 0.001 for 53% vs. 93%) (39).

A study from China reported 100% prevalence of *coo*, *Ig*, and *eno* genes in SCM isolates (33). In Brazil, researchers found a 93% prevalence for *fib* (31), while a survey in Thailand indicated a 96% prevalence for *pvl* (29), with all isolates in these studies originating from SCM cases. For CM, a study in China reported a 97% prevalence for *fnbA* (36), and researchers in India observed a 93% prevalence of *blaZ* (38). In the United States, high prevalences were reported for *hlgA* (99.2%), *hlgB* (100%), and *hlgC* (99.2%) (40). Additionally, analysis of 75 isolates from mixed SCM and CM cases in Iran revealed a prevalence of 97.3% for both *ebpS* and *fnb* genes (30). These findings highlight the geographical distribution and variability of virulence genes in *S. aureus* isolates associated with bovine mastitis.

#### The pathogenic mechanism of virulence factors of *Staphylococcus aureus*

In *S. aureus*, three categories of VFs involved in the infection process have been identified: adhesion factors (including *fnb*, *clfA/B*, *SdrD*, *IsdA*, and *ica*), biofilm-associated factors (including *ica* and *bap*), and toxins (including *hla*, *hlb*, and *hld*). Adhesion is the initial step in the IMI, allowing the pathogen to attach to host tissues. Biofilms formation enhances the survival of *S. aureus* in mammary tissue by protection from host defenses and antimicrobials (42). Toxins contribute to tissue damage and immune evasion by directly damaging host cells (43).

Colonization represents a pivotal phase in *S. aureus* pathogenesis, facilitated by a sophisticated repertoire of surface adhesins, biofilm-forming capacity, and toxin-mediated immune modulation (44, 45). The pathogen’s microbial surface components recognizing adhesive matrix molecules (MSCRAMMs), a family of cell wall-anchored proteins, play a central role in tissue-specific adhesion and immune evasion (46). Fibronectin-binding proteins A and B (FnBPA/B) are critical for invasion of the bovine mammary epithelial cell (BMEC), acting as molecular bridges that link host α5β1 integrins via fibronectin (47–49). While the *fnb* gene deletion attenuates adhesion, compensatory mechanisms involving other MSCRAMMs allow for continued colonization, highlighting functional redundancy in adhesion pathways (44, 50). Clumping factors A/B (*ClfA*/*B*), fibrinogen-binding adhesins, exemplify this adaptability. They can bind annexin A2 on BMECs independently of fibrinogen, thereby broadening host tissue tropism during early IMI (51–53). Additional adhesins, such as serine-aspartate repeat protein *SdrD* and iron-regulated surface determinant *IsdA* (dual fibrinogen/fibronectin binder), further diversify adhesion strategies and promote niche-specific colonization (54, 55).

Biofilm formation plays a critical role in *S. aureus* persistence within the mammary gland niche (42). The extracellular matrix, primarily composed of poly-*β*(1–6)-N-acetylglucosamine (PIA) synthesized by the *ica* operon, promotes adhesion to host cells and initiates biofilm development. In *ica*-deficient strains, biofilm-associated protein (Bap) compensates for the loss of PIA by interacting with the mammary epithelial Gp96 receptor, although this occurs at the expense of reduced invasiveness (44, 56, 57). Strains co-expressing *ica* and *bap* demonstrate enhanced biofilm complexity, underscoring the adaptability of *S. aureus* in different host environments (56).

Toxin production significantly contributes to tissue destruction and immune evasion (43). *α*-Hemolysin (Hla) binds to ADAM10 forming *β*-barrel pores that induce necrotic cell death through ion dysregulation. In addition, study have investigated the pathogenic mechanisms at the cellular and molecular levels. Specifically, bacterial toxins such as α-hemolysin directly impair host cystine uptake by downregulating SLC7A11, thereby inactivating the key antioxidant enzyme GPX4 and leading to uncontrolled lipid peroxidation (58), ultimately inducing cellular ferroptosis (59). And enterotoxin M activates the NF-κB pathway, exacerbates oxidative stress, and causes epithelial cell dysfunction (60). *β*-Hemolysin (Hlb) enhances this effect by hydrolyzing sphingomyelin in host membranes, thereby priming them for increased Hla cytotoxicity (61, 62). *δ*-Hemolysin (Hld) complements this lytic cascade by transiently disrupting membrane integrity and facilitating ion efflux through. Regarding the induction of cell apoptosis, investigations into lipoteichoic acid (LTA) have revealed that high concentrations of LTA increase the levels of nuclear receptor subfamily 4 group A (NR4A), which is involved in regulating downstream gene expression, thereby influencing apoptosis and necrosis (63). Notably, study on Panton-Valentine leukocidin (PVL) have demonstrated that this factor induces G0/G1 phase arrest and dephosphorylation of cell cycle-related proteins BCLAF1, CDK7, NF2, and PKM2 in a dose-dependent manner, thereby inhibiting bovine mammary epithelial cell (BMEC) proliferation (64). This multifactorial strategy, encompassing MSCRAMM-mediated adhesion, biofilm-mediated protection, and toxin-driven immune evasion, exemplifies the evolutionary adaptation of *S. aureus* in bovine mastitis. Targeting conserved virulence genes holds promise for disrupting pathogen colonization and survival.

### Streptococcus agalactiae

#### Prevalence of virulence genes in *Streptococcus agalactiae*

A total of 29 virulence genes were analyzed from 1,265 *S. agalactiae* isolates reported in studies from seven countries: China, India, Egypt, Argentina, Colombia, Pakistan, and Poland (Figure 3).

Among the analyzed genes, the surface protein gene *scpB* was examined in 812 isolates, including 291 from mixed SCM and CM and 521 from CM cases alone. The distribution of *scpB* showed considerable variability. All 521 CM samples were tested, but considerably different prevalence rates were reported in studies from China (7.0, 0.71, and 85%; *p* < 0.001 for 7.0% vs. 85%) (65–67). In contrast, moderate prevalence was observed in the 291 mixed SCM and CM from India (36%) (68), Poland (35%) (18), and Colombia (40%) (*p* < 0.001 for 40% vs. 85%) (69).

Hemolysin-related genes exhibited divergent prevalence patterns. The *cfb* gene (CAMP factor) was nearly ubiquitous in 764 samples from China (99 and 100%) (66, 70, 71), Poland (100%) (18), Pakistan (100%) (71), and Colombia (99%) (69). However, lower prevalence was observed in 57 additional samples from China (56%) and 42 samples from India (38%) (*p* < 0.001 for 56 vs. 99%) (65, 68). Similarly, the *hylB* gene (hyaluronidase) showed high prevalence (99–100%) in 1166 isolates from Egypt (72), Argentina (73), Colombia (69), Pakistan (71), China (66, 67, 70, 71), and Poland (18), but had a prevalence of 49% in the 57 samples from China (*p* < 0.001 for 49 vs. 99%) (65).

Adhesion-associated genes showed significant regional variability in prevalence. The *bca* gene (*α*-C protein) had a very low prevalence in 197 CM samples from China (2.1 and 5.3%) (65, 66) and similarly low levels in 68 mixed SCM and CM samples from Poland (8.8%, *p* = 0.44 for 8.8% vs. 5.3%) (18). In contrast, the prevalence was higher in 56 mixed SCM and CM samples from Argentina (36%, *p* < 0.001 for 36% vs. 8.8%) (73), this finding was also observed in a comparative study between China and Pakistan (23 and 24%) (71). The *cylE* gene (hemolysin transporter) was highly prevalent in 245 CM samples from China (97 and 100%), 270 mixed SCM and CM samples from China and Pakistan (100%), and in 68 mixed SCM and CM samples from Poland (96%) (18, 66, 70, 71), but had a significantly lower prevalence in 22 SCM samples from Egypt (68.2%, *p* < 0.001 for 68% vs. 96%) (72).

In addition, the *bac* gene was present in 100% in 22 SCM samples from Egypt (72), while the genes *fbsA*, *bibA*, *gapC*, and *dltA* had a prevalence of 98–100% in 140 CM samples from China (66). Among strains from mixed SCM and CM samples, *PI-2b* and *spb1* had a prevalence of 96% in Argentina (73), and *sip* and *cspA* in were detected on 100% of isolated from Poland (18).

#### Pathogenic mechanism of virulence factors of *Streptococcus agalactiae*

Regarding *S. agalactiae*, three categories of VFs involved in the infection process have been identified: adhesions (*fbs*, *lmb*, *bca*, and *sip*), toxins (*cfb*), and immune evasion factors (*hylB* and *cspA*). Functionally, adhesion represents the first step in in the IMI (74). Toxins contribute to pathogenicity by forming pores and exerting other damaging effects. Immune invasion factors involved help sustain the IMI by allowing the bacteria to avoid immune recognition and response (75).

Specifically, during bovine mastitis, *S. agalactiae* dynamically regulates a repertoire of VFs to adapt to the host microenvironment and evade immune clearance. Adhesion and invasion are mediated by fibrinogen-binding proteins (FbsA/FbsB), which anchor the pathogen to the host extracellular matrix (74), along with the laminin-binding protein (*Lmb*) and Bca protein, which facilitate epithelial attachment (76). The highly conserved surface immunogenic protein (Sip) not only promotes host cell penetration but also induces cross-protective immunity, underscoring its potential as a vaccine candidate. Pathogenicity is further enhanced by pore-forming toxins, including *β*-hemolysin and CAMP factor (encoded by *cfb*), which disrupt host cell membrane integrity. Notably, the CAMP factor plays dual roles as a cytolytic toxin and a species-specific diagnostic marker for streptococcal mastitis.

**Figure 4:**
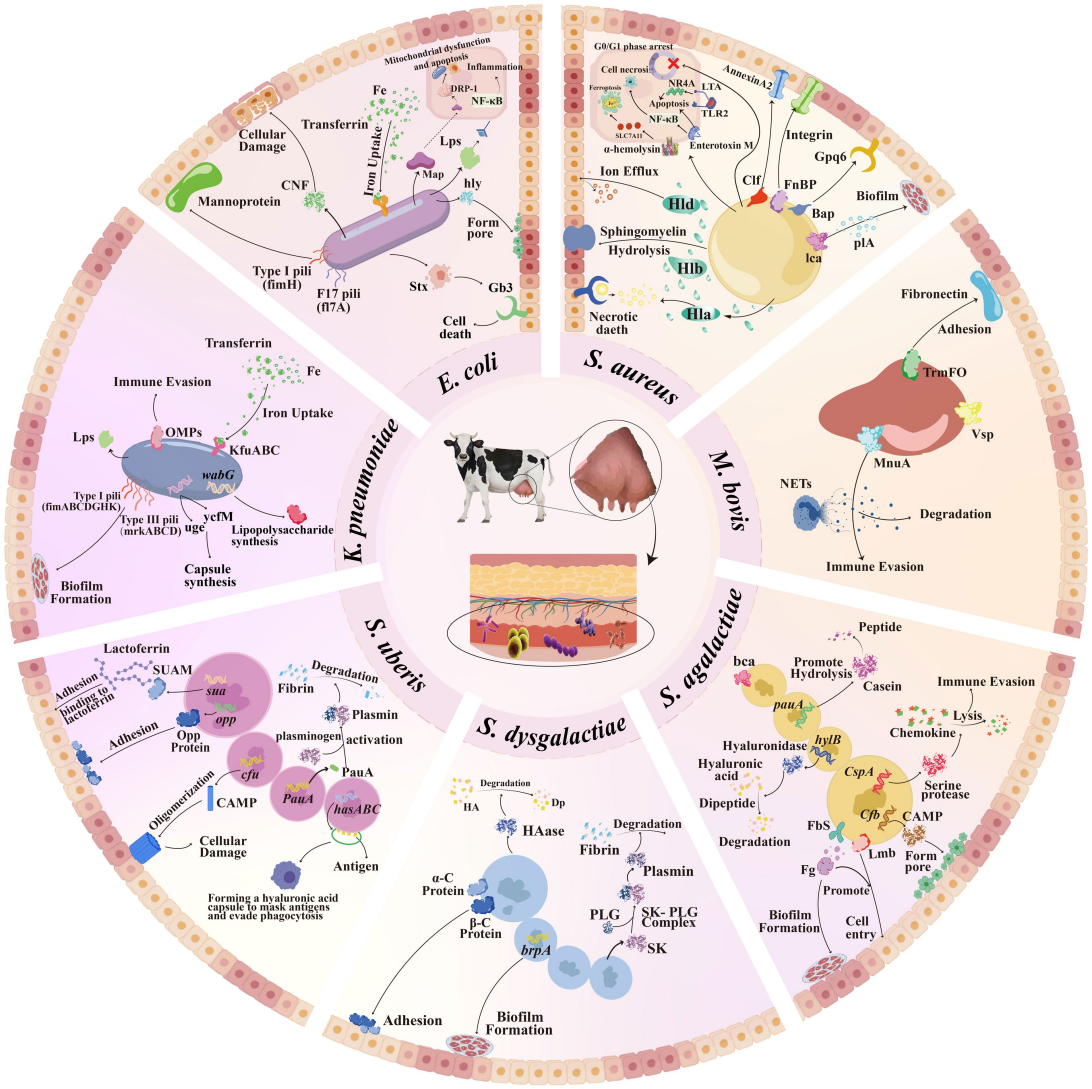
Schematic diagram of the pathogenic mechanism of virulence factors of bovine mastitis pathogens. *S. aureus*: FnBP promotes adhesion and invasion of mammary epithelial cells; Clf binds to the Annexin A2 receptor; PIA enhances adhesion; Bap binds to the Gp96 receptor; Hla induces tissue necrosis; Hlb hydrolyzes sphingomyelin to synergize with Hla; Hld disrupts membrane ion balance; *α*-hemolysin by downregulating SLC7A11, thereby inactivating the enzyme GPX4 and leading to ferroptosis; Enterotoxin M activates the NF-κB pathway, exacerbates oxidative stress, and causes epithelial cell dysfunction; LTA increase the levels of nuclear receptor subfamily 4 group A (NR4A), thereby influencing apoptosis and necrosis; PVL can induces G0/G1 phase arrest in cells, thereby inhibiting the proliferation of BMEC. *Mycoplasma* spp.: TrmFO binds to fibronectin to mediate adhesion; Vsp evades immune response through variation; MnuA degrades neutrophil extracellular traps. *S. agalactiae*: Fbs binds to fibrinogen to promote biofilm formation and assist Lmb in entering host cells; *bca* protein participates in adhesion; *cfb*-encoded CAMP factor forms cell pores; *hylB* hyaluronidase aids in invasion; *PauA* hydrolyzes casein; *cspA* protease cleaves chemokines. *S. uberis*: The protein synthesized by *sua* exerts adhesion by binding to lactoferrin; Opp protein also mediates adhesion; the CAMP factor encoded by the *cfu* gene forms pores in the host cell membrane, leading to cell damage. The PauA protein activates plasminogen to plasmin, causing degradation of the extracellular matrix, which in turn promotes the invasion of bovine mammary epithelial cells. The hyaluronic acid capsule encoded by the *hasA, hasB*, and *hasC* genes avoids phagocytosis by masking antigens. *K. pneumoniae*: Type I/III fimbriae are involved in biofilm formation; OMPs evade the immune response; *kfu* is involved in iron acquisition; *wabG* participates in the synthesis of lipopolysaccharides to resist phagocytosis; *uge/ycfM* enhances capsular resistance. *E. coli*: F17/Type I fimbriae (*f17A*/*fimH*) mediate adhesion; CNF induces cell necrosis and releases inflammatory factors; hemolysin forms membrane pores; iron transport system acquires iron; some strains produce Shiga toxin that binds to the Gb3 receptor to induce cell apoptosis; The effector protein Map induces apoptosis by triggering a decrease in mitochondrial membrane potential and DRP-1-dependent mitochondrial fission. *S. dysgalactiae*: α/β-C proteins mediate adhesion; *brpA* promotes biofilm formation; streptokinase activates plasmin to degrade tissue; hyaluronidase degrades hyaluronic acid to assist invasion. Created with BioGDP.com; reproduced with permission.

Immune evasion by *S. agalactiae* is achieved through multifactorial strategies. Hyaluronidase HylB (encoded by *hylB*) degrades hyaluronic acid into immunosuppressive disaccharides that inhibit Toll-like receptor signaling (77, 78), while also enhancing bacterial tissue invasion. The serine protease CspA (encoded by *cspA*) cleaves fibrinogen and chemokines, thereby impairing neutrophil recruitment and phagocytic activity (75). The Rib protein, characterized by domain atrophy due to the loss of core structural elements, likely evades immune recognition through molecular mimicry (75). Additionally, nutrient acquisition is facilitated via *pauA-*mediated hydrolysis of casein into amino acids, supporting bacterial proliferation (79) (Figure 4). These coordinated virulence mechanisms highlight the adaptability of *S. agalactiae* in response to host interactions. These findings emphasize that it is crucial to integrate the molecular characteristics of local pathogens when formulating regional prevention and control strategies. Targeting conserved VFs may provide novel strategies for the prevention and control of mastitis.

### Mycoplasma bovis

#### Prevalence of virulence genes in *Mycoplasma bovis*

The distribution of *Mycoplasma bovis* virulence genes has not been fully characterized, with differences in the prevalence of virulence genes reported between the two countries (Australia and Iran) (Figure 3). In 82 Australian isolates, the prevalence of the *clpC* gene, which encodes an ATP-dependent protease for proteostasis regulation, was 95%. The *tufA* gene, which encodes elongation factor Tu, was detected in 99% of the isolates, while the *MAG006* gene was present in 78%, with all three exceeding a 70% prevalence (80)‌. In contrast, among 21 Iranian strains, the detection rates of the *LppB* gene, which encodes lipoprotein, and the *P48* gene were 52.4 and 57.1%, respectively, both above 50% (81).

#### Pathogenic mechanism of virulence factors of *Mycoplasma bovis*

Three categories of VFs have been identified in the *Mycoplasma bovis* infection process: adhesion (e.g., TrmFO proteins), biofilm formation (e.g., Vsps), and immune evasion (e.g., *MnuA*). Functionally, adhesion represents the initial step in the IMI. Subsequently, biofilm formation and other immune evasion factors contribute to the persistence of IMI (82, 83).

Specifically, *M. bovis* utilizes a suite of membrane-bound VFs to colonize the host and evade immune defenses, taking advantage of its cell wall-deficient structure to enable direct host-pathogen interactions. Adhesion, a prerequisite for pathogenicity, is mediated by surface-exposed membrane proteins that bind to host extracellular matrix components. Among them, fibronectin (Fn), a key extracellular matrix glycoprotein, acts as a molecular bridge between bacterial adhesins and host cell receptors. TrmFO facilitates adhesion by binding to Fn (84–86). Furthermore, anti-rTrmFO antibodies can inhibit the adhesion of *M. bovis* to cells, thereby reducing virulence. This underscores the crucial role of TrmFO-mediated adhesion in the infection process (86).

Biofilm formation in *M. bovis* IMI is regulated by variable surface lipoproteins (Vsps), which serve as a major structural component interacting with the host. Through structural variation, Vsps evade immune recognition, thereby enabling persistent colonization (82, 87). Simultaneously, immune evasion is enhanced by the membrane-associated nuclease MnuA, which degrades neutrophil extracellular traps (NETs), allowing the pathogen to resist phagocytic killing (83). In addition, *M. bovis* secretes effector proteins such as the lipoprotein MbovP475, which binds to promoter regions of host cell cycle regulators in bovine macrophages, suppressing their transcriptional activity and impairing immune function (88, 89) (Figure 4).

These coordinated mechanisms, including dynamic surface antigen variation, biofilm adaptability, NET degradation, and modulation of host gene expression, position *M. bovis* as a stealth pathogen capable of subverting both innate and adaptive immune responses. Targeting conserved adhesion machinery or immunoreactive VFs may provide novel therapeutic avenues for the prevention and treatment of bovine *Mycoplasma* mastitis.

### Escherichia coli

#### Prevalence of virulence genes in *Escherichia coli*

A total of 55 virulence genes were investigated across 1,115 *E. coli* isolates collected from 11 countries, including Brazil, Switzerland, China, Egypt, Pakistan, Iran, Turkey, Ireland, Vietnam, South Korea, and Jordan (Figure 3).

The *α*-hemolysin gene (*hlyA*) was detected in 330 isolates, showing an overall low prevalence (<20%). It was particularly rare in China (8%) (90) and Brazil (7%) (91), while slightly higher rates were observed in Iran (19%) (92) and Switzerland (18%, *p* = 0.048 for 18% vs. 8%) (93).

Adhesion-related genes show complex distribution across regions. The reported prevalence of the *fimH* gene is considerably higher in Brazil compared to other regions, reaching 100 and 94% in 224 CM samples (91, 94). In contrast, the prevalence is slightly lower in isolates from China (90%) (95), South Korea (81%) (96), and Egypt (80%) (*p* = 0.35 for 90% vs. 94%, *p* = 0.07 for 90% vs. 81%) (97). The prevalence of the *traT* gene showed high prevalence (>60%) across 379 CM samples and 82 mixed CM and SCM samples, 77 and 82% in Brazil (91, 94), 72% in Switzerland (93), and 67% in Turkey (98). However, its prevalence was notably lower in 300 mixed SCM and CM, and packaged milk samples from China, Pakistan, and South Korea, 46% in China (90), 27% in Pakistan (99), and 27% in South Korea (96) (*p* = 0.002 for 46% vs. 67%).

Iron acquisition-related genes showed considerable variability in prevalence across regions. The *iucD* gene had the highest prevalence in Pakistan, where it was detected in 30% of mixed CM and SCM samples (99). In contrast, it was considerably lower among CM (*n* = 110) and SCM (*n* = 70) samples in Iran (*n* = 70 SCM) and Brazil (*n* = 110 CM), both reporting a prevalence of 4% (*p* < 0.001 for 4% vs. 30%) (94, 100). Additionally, two studies from China reported different results: *iucD* was detected in only 1% of 79 CM samples, compared to 10% in 87 from herds with both SCM and CM (*p* = 0.01 for 10% vs. 1%) (90, 95). Similarly, the prevalence of the *irp2* gene varied within and between countries. In Brazil, it was reported at 10 and 35% among a total of 224 CM samples (91, 94). In China, the prevalence was lower, at 3 and 13% in 166 CM and mixed CM and SCM samples in China, respectively (*p* < 0.001 for 13% vs. 35%) (91, 95).

Additionally, several other highly prevalent genes have been identified. One study reported a 100% prevalence of the *tetB*, *tetE*, *tetG*, and *ereA* genes among 14 CM samples in Jordan (101). Another study found a 100% prevalence of the *Aer* and *ompA* genes among 87 mixed CM and SCM isolates in China (90).

#### Pathogenic mechanism of virulence factors of *Escherichia coli*

Four main categories of VFs involved in the infection process with *E. coli* have been identified: adhesion (e.g., *f17*, *lpf*, and *fimH*), biofilm formation (e.g., *ecpA*), toxins (e.g., *hly* and *Stx*), and iron acquisition (e.g., the Fec system, *irp2*, and *iucD*). Functionally, adhesion factors facilitate the initial attachment to the mammary tissue, while biofilm formation helps sustain the IMI (102). Tissue damage is primarily mediated by toxins, whereas bacterial survival depends on immune evasion and iron uptake (103).

Specifically, although the bovine mammary gland is not a primary habitat for *E. coli* due to innate immune defenses, such as antimicrobial peptides, lysozymes, and the complement system (104), certain virulent strains can overcome these barriers by employing adhesion factors, toxins, and iron acquisition systems to establish IMI (105, 106).

Adhesion is mediated through interactions with host fibrinogen and laminin receptors, facilitated by both fimbrial and non-fimbrial adhesins. The F17 pili (*f17A*-encoded) and long polar fimbriae (LPF1/LPF2, *lpf*-encoded) are critical for the colonization and invasion of bovine mammary epithelial cells (107–109). Additionally, the high prevalence of *ecpA* (essential for biofilm initiation) and *fimH* (which mediates mannose-specific binding via type 1 pili) underscores their roles in sustained attachment (91, 102). In addition, at the intracellular molecular level. Research has demonstrated that the secreted effector protein Map targets mitochondria in host bovine mammary epithelial cells, ultimately inducing apoptosis by triggering a decrease in mitochondrial membrane potential and DRP − 1-dependent mitochondrial fission (110). This process has been further confirmed to rely on the ERK signaling pathway, and the pro-apoptotic effect of Map in this pathway is independent of another effector protein, EspF (111).

Cytolytic exotoxins contribute significantly to tissue damage, *α*/*β*-hemolysin (*hly*-encoded) forms pores in host membranes, whereas cytotoxic necrotizing factors (CNF1/CNF2) induce cell necrosis and stimulate the release of pro-inflammatory cytokines thereby exacerbating local inflammation (19). Lipopolysaccharide (LPS), a major component of the outer membrane, further amplifies the inflammatory response by upregulating proinflammatory mediators such as TNF-α, IL-6, and IL − 1β in mammary tissues (112). Beyond direct cellular damage, the structure of the LPS O-polysaccharide chain plays a crucial role in immune evasion, its smooth configuration attenuates TLR4/NF-κB-mediated inflammatory responses and endows bacteria with resistance to complement-mediated killing, thereby facilitating their survival in the mammary gland microenvironment (105). Additionally, certain *E. coli* strains produce Shiga toxins (Stx), which are classified into two major subtypes: Stx1 and Stx2. These toxins bind specifically to glycosphingolipid receptors, such as globotriaosylceramide (Gb3), on host cell membranes. Upon receptor-mediated endocytosis, Stx exerts its cytotoxic effect by catalytically inhibiting ribosomal protein synthesis, ultimately triggering apoptotic cascades in epithelial cells (Figure 4) (113, 114).

Iron acquisition is critical for *E. coli* proliferation in the iron-limited environment of milk. The ferric citrate (Fec) transport system is essential for virulence, as demonstrated by the inability of Fec-deficient P4 strains to induce mastitis, whereas Fec acquisition enables otherwise non-pathogenic K71 strains to successfully infect the mammary gland (103, 115). This reliance on Fec may explain the low prevalence of alternative iron-scavenging genes, such as *irp2*, *iucD*, in mastitis-associated isolates (116).

Collectively, these coordinated strategies, including adhesion, toxin-mediated damage, and iron piracy, highlight *E. coli*’s adaptability in overcoming host mammary defenses.

### Streptococcus uberis

#### Prevalence of virulence genes in *Streptococcus uberis*

A total of 12 virulence genes from 487 *S. uberis* isolates collected in the Czech Republic, Thailand, Argentina, Egypt, Brazil, and China were included (Figure 3).

The adhesion-related genes exhibited country-specific prevalence patterns. The *sua* gene was detected in the 85 CM samples and 402 mixed SCM and CM samples from the above-mentioned six countries, and its overall prevalence was relatively high (71–100.0%) except in Egypt (117–122). In the 69 CM samples from Egypt, the detection rate was 42%, which was relatively lower (*p* < 0.001 for 42% vs. 71%) (118). Similarly, the *pauA* gene also showed similar geographical differences. It was detected in samples from the six countries, with a relatively high overall carriage rate (59–97%) except in Egypt (117–122). In the 69 CM samples from Egypt, the detection rate was 39%, which was relatively lower (*p* = 0.01 for 39% vs. 59%) (118). In contrast, the *lbp* gene showed considerable geographic variation, with a higher prevalence of 63% in 88 mixed SCM and CM samples in Thailand (121). Still, lower prevalence was observed in 269 mixed SCM and CM samples and 16 CM samples from China, Argentina, and the Czech Republic, at 25, 12, and 2.1%, respectively (*p* = 0.005 for 25% vs. 63%) (117, 119, 122).

The gene encoding CAMP factor (*cfu*) was reported in 85 CM strains and 356 mixed SCM and CM samples from five countries, excluding Brazil, with prevalence of 56% in China (119) and 77% in Argentina (122). In contrast, the detection rates were relatively lower in the 278 mixed SCM and CM samples from the Czech Republic and Thailand, and the 69 CM samples from Egypt, at 5.8% (117), 28% (121), and 22% (118), respectively (*p* = 0.03 for 28.4% vs. 56.3%).

Additionally, several other commonly detected genes have been reported. In studies from the Czech Republic, the prevalence of *hasA*, *hasB*, *skc*, *gapC*, and *oppF* among 190 mixed SCM and CM samples ranged from 94 to 100% (117). The *gapC* gene was predominant in 16 CM samples from China, with a prevalence of 100% (119).

#### Pathogenic mechanism of virulence factors of *Streptococcus uberis*

Several major virulence factors (VFs) have been identified during the *S. uberis*, infection process, including adhesion, immune evasion, and hydrolases. Functionally, *S. uberis* initiates IMI through adhesion, maintains IMI by evading immune recognition (76), and promotes tissue degradation through the action of hydrolases (123).

As a CAMP test-positive pathogen like *S. agalactiae* (124), *S. uberis* primarily colonizes the ducts and alveolar spaces, rarely invading mammary tissue; so the corresponding gene products may be important for the growth or survival of *S. uberis* within the bovine mammary gland (125). *Streptococcus uberis* colonizes bovine mammary tissue by utilizing a variety of virulence mechanisms. These mechanisms include adhesion, immune evasion, and enzymatic tissue degradation, demonstrating the adaptability of *S. uberis* in maintaining IMI within the host. Specifically, internalization of *S. uberis* into mammary epithelial cells is an early event in the occurrence of bovine mastitis. In this context, the *Streptococcus uberis* adhesin molecule (SUAM) encoded by the *sua* gene is speculated to have affinity for host lactoferrin, which appears to promote pathogenesis. Lactoferrin is a protein present in bovine milk and many other mammalian body fluids (126), and lactoferrin may act as a bridging molecule for *S. uberis*, thereby facilitating adhesion and internalization into mammary epithelial cells, thus surviving in host defense mechanisms through immune evasion (127). However, other studies have demonstrated that certain factors overlap in function with *sua*. Similar to *sua*, deletion of the *vru* gene reduced the virulence of *S. uberis*, possibly due to its ability to bind lactoferrin (128). The capsular polysaccharides encoded by the *cps* and *neu* genes evade the immune system by masking pro-inflammatory cell wall components. The C5a peptidase encoded by *scpB* is another virulence factor that leads to immune evasion. C5a is a component of the human complement system, and its degradation results in the inhibition of the opsonophagocytic killing pathway. In addition, peptidases have been shown to bind fibronectin, thereby contributing to bacterial adhesion and invasion in epithelial cells (76).

The evolving understanding of the *hasABC* operon in *S. uberis* highlights the complexity of bacterial virulence. An early study emphasized the role of the hyaluronic acid capsule in anti-phagocytosis, proposing that the *hasABC* genes are crucial for virulence (129). The operon is a conserved gene region that can produce a hyaluronic acid capsule, protecting bacteria from opsonization and phagocytosis, and mediating resistance to bacterial clearance within neutrophil extracellular traps (123). However, subsequent studies have demonstrated the existence of compensatory pathways that may maintain capsule-independent infection (130). This suggests that *S. uberis* may adapt to different environmental stresses through different genetic mechanisms (131).

It is worth noting that studies have found that the *cfu* gene encoding the CAMP factor is present in strains causing transient mastitis. This factor oligomerizes to form tubular structures, creating pores in the host cell membrane and ultimately leading to cell damage (131). However, it is important to note that research on *S. uberis* in the mammary environment is relatively limited. Therefore, further in-depth studies are still needed (Figure 4).

### Klebsiella pneumoniae

#### Prevalence of virulence genes in *Klebsiella pneumoniae*

A total of 24 virulence genes were analyzed of 1,104 *K. pneumoniae* isolates from China, India, Egypt, and the United States (Figure 3).

The prevalence of the iron acquisition gene *entB* ranged from 78 to 97% among 794 isolates in China (132–134), higher than the 21% detected in 180 isolates in the United States (*p* < 0.001 for 21% vs. 78%) (135). The prevalence of *kfu* was relatively high in Egypt (80.0%) and in one study from China (62.9%) (132, 136), but considerably lower in two additional Chinese studies, with reported prevalence of 31 and 36%, respectively (133, 134).

Capsular polysaccharide regulation showed extreme contrasts: the prevalence of *rpmA* was 80% among 35 samples in Egypt (136), compared to 4.4% among 499 samples in China (*p* < 0.001 for 4.4% vs. 80%) (132, 137).

Additionally, several other highly prevalent genes were detected in 68 CM samples from China, with *wabG*, *fimH1*, *uge*, and *ureA* genes each detected at a prevalence of 100% (137).

#### Pathogenic mechanism of virulence factors of *Klebsiella pneumoniae*

Four main categories of VFs have been identified in the infection process with *K. pneumoniae*: adhesion (e.g., *fimABCDGH*, *fimK*, and *mrkABCD*), biofilm formation (e.g., *omp* and *uge*), toxins (e.g., *wabG*), and iron acquisition (e.g., *kfu*). Functionally, *K. pneumoniae* initially adheres to host cells via adhesion factors and establishes persistent colonization through biofilm formation (138, 139). Tissue damage is caused by toxins (140), while proliferation is supported by iron acquisition mechanisms (141).

Specifically, *K. pneumoniae* employs a multifaceted arsenal of VFs to colonize and damage bovine mammary tissues. Adhesion, a critical initial step to IMI, is mediated by type I fimbriae (encoded by *fimABCDGH*), which facilitate host cell binding. *fimK* also contributes to this process, playing an essential, though not yet completely characterized, role in fimbrial assembly and adhesion efficacy (138, 142, 143). Type III fimbriae (encoded by *mrkABCD*) further enhance biofilm formation and persistent colonization (139). Surface structures such as capsular polysaccharide (CPS) and outer membrane proteins (OMPs, encoded by *omp*) act synergistically to promote immune evasion by masking bacterial antigens and resisting phagocytosis (19). The endotoxin-associated gene *wabG* is mechanistically linked to the biosynthesis of core lipopolysaccharide in the outer membrane, a critical virulence determinant that enhances bacterial evasion of macrophage phagocytosis (140). Furthermore, VFs such as *uge* (uridine diphosphate galactose/glucose-4-epimerase) and *ycfM* (outer membrane lipoprotein) are functionally associated with the biosynthesis of the polysaccharide capsule. These components collectively mediate resistance to phagocytosis by structural interference with opsonophagocytic recognition, thereby potentiating systemic infection (Figure 4) (144).

Iron acquisition is pivotal for bacterial proliferation in the iron-restricted environment of the mammary gland. Clinical mastitis isolates harboring siderophore genes exhibit demonstrate enhanced growth under iron-depleted conditions, with the *kfu* operon playing a key role in iron uptake and disease pathogenesis (141, 145). Additionally, bovine *K. pneumoniae* mastitis strains frequently carry lactose operons (*lac*), which may enhance metabolic fitness in lactose-rich mammary tissues (146). Collectively, these mechanisms, including adhesion, biofilm formation, and enzymatic tissue degradation, demonstrate the adaptability of *K. pneumoniae* in sustaining infection within the host.

### Streptococcus dysgalactiae

#### Prevalence of virulence genes in *Streptococcus dysgalactiae*

A total of 63 virulence genes were analyzed of 155 *S. dysgalactiae* isolates from in China, the United States, and Portugal (Figure 3).

Surface protein genes exhibited country-specific prevalence patterns. The *scpB* gene was detected exclusively in 60 CM and 23 mixed SCM and CM samples from China, though its overall prevalence was low (15–20.0%, *p* = 0.772 for 15% vs. 20.0%) (147–149). In contrast, the *lmb* gene showed significant geographic variation, with a high prevalence in 38 CM samples from the United States (65.8%), compared to lower but variable prevalence in 83 isolates from China (3.3, 15.4, and 60.0%; *p* = 0.072 for 15.4% vs. 60.0%) (147–150).

The adhesion-related *α*-C protein gene (*bca*) was reported in 60 CM and 23 mixed SCM and CM samples from China, with detection in 13 and 10 isolates, respectively (prevalence 6.7–20.0%) (147–149).

Additionally, several other commonly detected genes have been reported. In studies from the United States, the prevalence of *perR*, *leus*, *gldA*, and *purH* among 35 CM samples ranged from 91.4 to 100.0% (150). The *sagA* gene was predominant in 37 mixed SCM and CM samples from Portugal, with a prevalence of 100% (151).

#### Pathogenic mechanism of virulence factors of *Streptococcus dysgalactiae*

Regarding *S. dysgalactiae*, three main categories of VFs have been identified in the infection process: adhesion, biofilm formation, and hydrolytic enzymes. Functionally, *S. dysgalactiae* initiates IMI through adhesion, sustains the infection via biofilm formation (152), and facilitates tissue degradation and bacterial dissemination through the action of hydrolytic enzymes (153).

Specifically, as a CAMP test-negative pathogen distinct from *S. agalactiae* (124), *S. dysgalactiae* employs multifaceted virulence mechanisms to colonize bovine mammary tissues. These mechanisms, including adhesion, biofilm formation, and enzymatic tissue degradation, demonstrate the adaptability of *S. dysgalactiae* in sustaining infection within the host. Critical to its pathogenicity are the *α*- and *β*-C proteins, which cooperatively mediate bacterial adhesion to host epithelial cells. The α-C protein, firmly anchored to the bacterial cell wall, facilitates robust attachment to host surfaces while modulating immune evasion through complex host-pathogen interactions. Synergistically, the β-C protein enhances bacterial invasiveness and overall pathogenicity (154, 155).

Biofilm formation, a key virulence trait of *S. dysgalactiae*, is driven by its ability to bind host extracellular matrix proteins. Biofilm formation, a key virulence trait of *S. dysgalactiae*, is driven by its ability to bind host extracellular matrix proteins. Certain strains demonstrate biofilm-forming capacity on hydrophilic surfaces, with the *brpA* gene implicated in early biofilm establishment (152). Additionally, tissue penetration is facilitated through plasminogen activation: streptokinase secreted by *S. dysgalactiae* converts plasminogen to plasmin, enabling degradation of connective tissue proteins (153). This proteolytic activity is further complemented by hyaluronidase-mediated cleavage of hyaluronic acid, promoting bacterial dissemination (Figure 4).

## Vaccine therapy targeting virulence factors of mastitis pathogens

Contagious pathogens such as *S. aureus*, *S. agalactiae*, and *M. bovis* predominantly induce subclinical IMIs causing SCM through cow-to-cow transmission. In contrast, environmental pathogens such as *E. coli* and *K. pneumoniae* are more commonly associated with CM (24, 156). Traditional therapeutic approaches for these IMIs involve the use of antimicrobials. However, the growing problem of antimicrobial resistance (AMR) has highlighted significant limitations of these methods. The widespread and often indiscriminate use of broad-spectrum antibiotics promotes the selection of resistant strains, such as methicillin-resistant *S. aureus* (MRSA) and extended-spectrum *β*-lactamase (ESBL)-producing Enterobacteriaceae, while also disrupting the commensal microbiota. This disruption can lead to dysbiosis and increased risk of secondary infections (157–159).

In studies on AMR, a total of 53 antimicrobials have been evaluated across different countries. Resistance among mastitis pathogens has been reported for several major antimicrobial classes, including β-lactams, aminoglycosides, fluoroquinolones, tetracyclines, and sulfonamides (160). All six pathogens included in this study demonstrated resistance to different antimicrobials (Table 1). Notably, AMR rates for drugs such as amikacin, oxytetracycline, and meropenem exceeded 50% in some countries. For detailed results, please refer to the Supplementary material 3.

**Table 1:** Phenotypic resistance of major bovine udder pathogens to some antimicrobials.

Antibiotic	Resistance rate						
	*S. aureus* (32, 180–192)	*S. agalactiae* (4, 71, 181–183, 189, 192–196)	*M. bovis* (197, 198)	*E. coli* (100, 101, 178, 180–183, 199–203)	*K. pneumoniae* (137, 145, 180, 204–206)	*S. uberis* (118, 119, 196, 207–213)	*S. dysgalactiae* (147, 182, 183, 196, 214, 215)
Streptomycin	17–100%	62–100%	-^1^	0-100%	26–41%	87–97%	28–100%
Gentamicin	0–72%	5–100%	-^1^	0-80%	4–77%	0–96%	11–78%
Erythromycin	8–85%	1–100%	-^1^	89.47%	-^1^	4–74%	22–100%
Ceftazidime	0%	0%	-^1^	0–16%	2%	-^1^	0%
Kanamycin	-^1^	0–96%	100%	21–32%	10–100%	30–83%	0–90%
Cefotaxime	0–40%	14%	-^1^	3–23%	3–33%	-^1^	45%
Neomycin	0-46%	0–80%	-^1^	0–32%	-^1^	0–52%	0–52%
Oxytetracycline	8–75%	77%	100%	71%	-^1^	93%	90%
Ampicillin	5-65%	0–37%	-^1^	0–90%	11.3%	1–90%	15%
Tetracycline	5–83%	13–100%	-^1^	0–90%	6–40%	1–86%	61–100%

^1^“-” = not tested.

**Figure 5:**
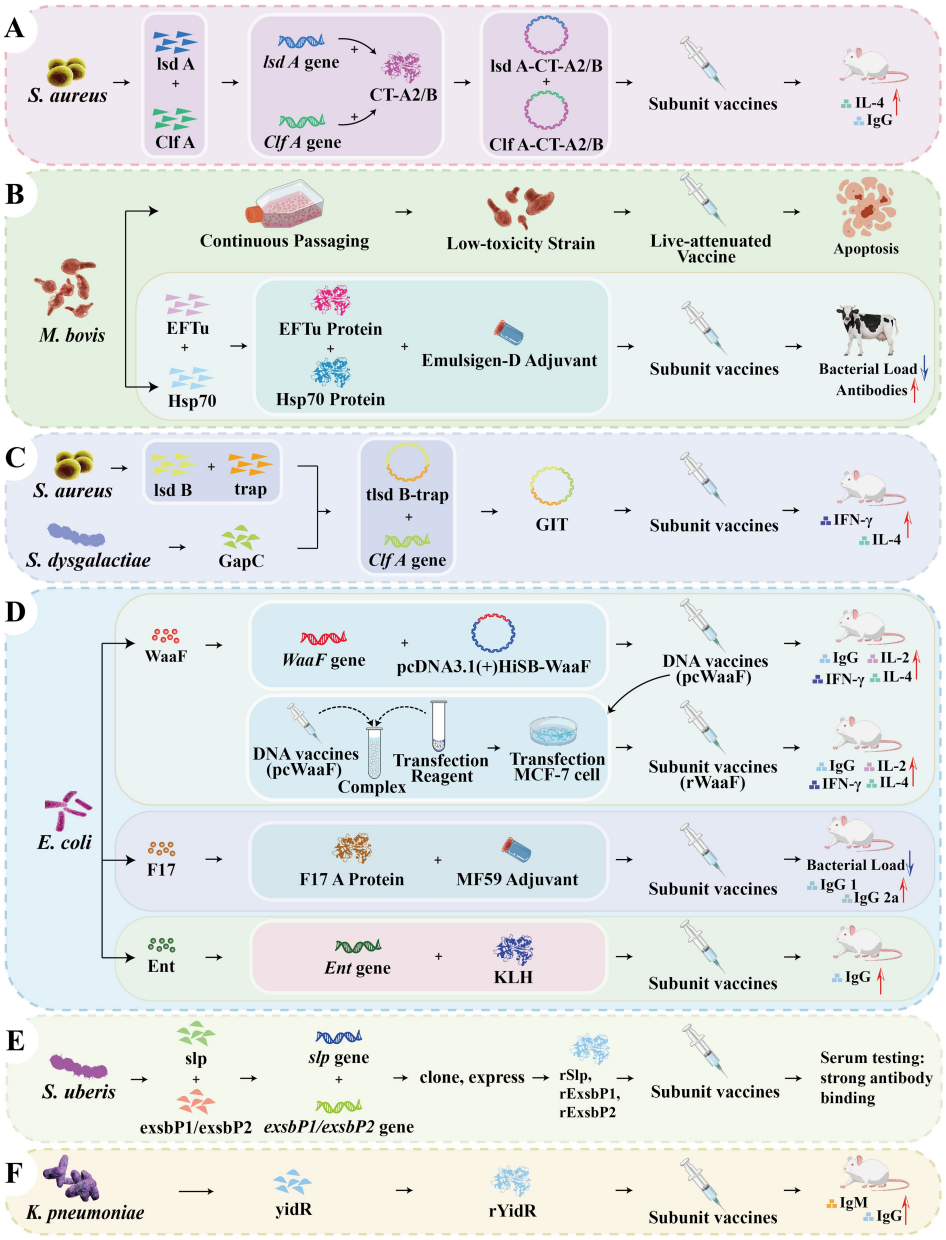
Schematic diagram for the development of vaccines targeting certain pathogens. **(A)** In the vaccine development against *S. aureus*, *IsdA* and *ClfA* were each substituted for the A1 domain of cholera toxin to construct two fusion proteins, IsdA-CTA2/B and ClfA-CTA2/B, completing the vaccine construction. **(B)** For the vaccine research against *M. bovis*, attenuated vaccine strains were obtained by continuous *in vitro* passage of wild strains. In another study, conserved bacterial proteins (EFTu and Hsp70) were used as antigens to prepare vaccines in combination with adjuvants. **(C)** A multivalent chimeric vaccine against *S. aureus* and *S. dysgalactiae*. The truncated GapC (*GapC1*) from *S. dysgalactiae* and the truncated *IsdB* from *S. aureus* were fused with TRAP. The genes were amplified and cloned into an expression vector to construct the GIT chimeric protein for vaccine development. **(D)** For the development of vaccines against *E. coli*, the *waaF*, *F17*, and *Ent* genes were targeted. The *waaF* gene was inserted into a vector to construct a recombinant plasmid. Subsequently, the *waaF* gene was inserted into the eukaryotic expression vector pcDNA3.1 to create a DNA vaccine (pcwaaF). The pcwaaF plasmid DNA was transfected into MCF-7 cells to obtain the recombinant WaaF protein for the construction of a subunit vaccine (rWaaF). The *F17* gene was cloned into the pET28a expression vector. The purified F17A protein was mixed with the MF59 adjuvant to prepare a subunit vaccine. The iron carrier *Ent* was conjugated with *KLH* (keyhole limpet hemocyanin) to prepare the *KLH*-*Ent* conjugate vaccine. **(E)** In the vaccine development study against *S. uberis*, the genes *slp*, *exsbP1*, and *exsbP2* were cloned into an expression vector. They were then transformed into *E. coli*. The expressed and purified recombinant proteins were used as antigens to complete the vaccine construction. **(F)** In a vaccine development study against *K. pneumoniae*, the *YidR* gene was cloned into an expression vector. The expressed and purified YidR protein was mixed with 20% aluminum hydroxide adjuvant to complete the vaccine construction. Created with BioGDP.com; reproduced with permission.

In light of these concerns, current control of mastitis should emphasize the prudent use of antimicrobial agents to minimize antimicrobial residues and the development of AMR (161). As alternatives to conventional antimicrobial therapies, immune-related therapies, such as vaccine development, are actively being explored (Figure 5).

An important aspect of therapies targeting VFs is vaccine development, which includes subunit, DNA, chimeric, and attenuated platforms aimed at combating pathogens like *E. coli*, *S. aureus*, *K. pneumoniae*, *S. uberis*, and *M. bovis*. These strategies utilize pathogen-specific VFs to design targeted immunotherapies, offering promising avenues for disease prevention and control.

### Staphylococcus aureus

*Staphylococcus aureus*, a predominant contagious udder pathogen, requires innovative approaches to address its immune evasion mechanisms. One such approach involves constructing fusion vaccines by replacing the toxic A1 domain of cholera toxin with *S. aureus* VF *IsdA* (iron-regulated surface determinant) and *ClfA* (clumping factor A), resulting in two chimeric proteins: *IsdA*-*CTA2/B* and *ClfA-CTA2/B* (Figure 5A) (162). These chimeric proteins elicited opsonic antibodies and Th2-polarized cellular immune responses in clinical studies, with *in vitro* assays confirming enhanced phagocytic activity against *S. aureus*. While clinical trials are still pending, these candidates represent a strategic shift toward toxoid-antigen hybrid vaccines. To broaden the spectrum of protection, a separate chimeric vaccine was developed by combining a truncated *S. dysgalactiae GapC* (GapC1) with truncated *S. aureus* truncated *IsdB* and TRAP (thrombin-sensitive protein) (163). The corresponding gene fragments (GapC1 and tIsdB-TRAP) were cloned into pET-32a (+), expressed in *E. coli*, and purified using Ni-NTA resin (Figure 5C). The GIT (GapC1-tIsdB-TRAP) vaccine induced cross-protective immunity in mice against both *S. aureus* and streptococci, demonstrating its potential as a broad-spectrum solution.

### Mycoplasma bovis

*Mycoplasma bovis* is a cell wall-deficient pathogen. Through serial *in vitro* passage over 150 generations, the wild-type P1 strain was attenuated to produce the P150 variant, which retained immunogenicity while exhibiting reduced virulence. Notably, it was found to upregulate CHOP (C/EBP homologous protein), triggering apoptosis in BoMac cells, a mechanism linked with its attenuation. This discovery not only elucidates P150’s safety profile but also offers valuable insight for future live vaccine development, suggesting that targeting apoptotic pathways may help balance efficacy and safety (164). Another study used conserved bacterial proteins (EFTu and Hsp70) as subunit vaccine antigens, combined with Emulsigen-D adjuvant to prepare the vaccine. After challenge with *M. bovis*, the vaccinated group produced high-titer antibodies, and T cells proliferated significantly in response to both antigens. Improvements were observed in bacterial load and pathological changes. However, the vaccine induced partial protection in American bison and reduced lung lesions, but its efficacy in specifically preventing bovine mastitis is still unclear. Future directions should focus on optimizing adjuvants, increasing antigens, or combining with other vaccine components to enhance protective efficacy (165) (Figure 5B).

### Escherichia coli

*Escherichia coli* establishes infection through lipopolysaccharides (LPS), adhesins, and iron chelation systems. In response, researchers have developed several subunit vaccine candidates targeting these virulence mechanisms. One such approach involves an LPS-based DNA vaccine. The *waaF* gene which encodes glycosyltransferase II (an enzyme involved in LPS synthesis), was first cloned into the pGEM-T-easy vector and then inserted into the pcDNA3.1 expression vector to construct the DNA vaccine pcwaaF. Upon transfection into MCF-7 cells, the recombinant waaF protein was successfully expressed, demonstrating both safety and immunogenicity (166) (Figure 5D). To block bacterial adhesion, the *f17a* gene, encoding the structural protein of the F17 fimbriae, was cloned into the pET28a vector and expressed in *E. coli*. The purified F17A protein was combined with the MF59 adjuvant to formulate a subunit vaccine, which significantly reduced bacterial adhesion and showed protective efficacy in experimental models (167) (Figure 5D). For iron restriction inhibition, a siderophore-based strategy was employed. The siderophore enterobactin (*Ent*) was purified from the *E. coli* mutant strain (AN102) and conjugated with keyhole limpet hemocyanin (KLH) to generate the KLH-Ent vaccine, This formulation induced specific antibodies in cows, effectively limiting bacterial iron uptake (168) (Figure 5D). Together, these approaches offer promising strategies for preventing of *E. coli*-induced mastitis.

### Streptococcus uberis

Subunit candidate vaccines have been developed to control *S. uberis* IMI. In one study, *S. uberis* was co-cultured with bovine mammary epithelial cells. Based on the prediction of immunogenic epitopes, three genes, *slp*, *exsbP1*, and *exsbP2*, were selected. The genes were cloned into the pET101/D-TOPO® expression vector and then transformed into *Escherichia coli*. The recombinant proteins were expressed by induction with IPTG. A subunit vaccine was made using purified rSlP + rExsbP1 + rExsbP2 as antigens. After challenge with the bacteria, all three recombinant proteins were recognized and strongly reacted with the serum from recovered cows, confirming their immunogenicity. They can be used as candidate antigens for vaccines to prevent or control bovine mastitis caused by *S. uberis* (169) (Figure 5E).

### Klebsiella pneumoniae

*Klebsiella pneumoniae* has been targeted by researchers through the development of a recombinant vaccine based on the YidR protein. YidR is a conserved antigen found across mastitis-associated *K. pneumoniae* strains. The gene encoding YidR was cloned into the pET6xHis/6his-yidR vector, expressed in *E. coli*, and formulated with aluminum hydroxide (Alhydrogel) as an adjuvant to produce the rYidR vaccine. This vaccine demonstrated strong protective efficacy in a mouse model, evidenced by the induction of high antibody titers, improved survival rates, and reduced clinical symptoms such as sepsis and weight loss (170). Importantly, the rYidR vaccine also helped maintain milk production in cases of *E. coli* CM (171), highlighting the broader utility of phylogenetically stable antigens for controlling Gram-negative mastitis (Figure 5F).

The advances in vaccine development against VFs (mainly subunit vaccines) have yielded promising results, and future research should focus on further development and validation. For example, field trials are needed to evaluate the iron-restriction efficacy of the KLH-Ent vaccine across diverse dairy herds. Similarly, the cross-protective potential of the GIT vaccine against *S. aureus* should be confirmed in dairy cows under real-world conditions. In addition, optimizing adjuvants and delivery systems based on the physiological status of individual cows could enhance vaccine potency and extend the duration of protection potency.

## Discussion

There are significant geographical variations in the prevalence of virulence genes among pathogenic bacteria associated with bovine mastitis, and these variations are closely correlated with clinical manifestations. For instance, many genes, including *clfA* and *clfB* have only been detected in research samples derived from subclinical mastitis or mixed mastitis (both CM and SCM) (29–35). In contrast, none of these genes have been reported in studies where all research samples were obtained from clinical mastitis cases (36–38). This indicates that differences in gene functions can indeed lead to distinct clinical outcomes. Furthermore, for the same gene sourced from identical mastitis types (the *clfA* gene isolated from subclinical mastitis), the reported detection rate was only 22% in Ethiopia, whereas the lowest detection rate among the other four countries with positive findings (China, Brazil, Thailand, and Iran) reached 77%. This also demonstrates that regional disparities can affect the prevalence of virulence genes. Such variations may be influenced by husbandry management practices and antimicrobial usage patterns. Specifically, in regions characterized by intensive farming systems and a long history of antibiotic application, pathogenic bacteria may be subjected to stronger selective pressures. This consequently leads to an increased prevalence of virulence genes associated with biofilm formation, immune evasion, and other virulence-related processes. For example, the prevalence of *Ig* genes with immune evasion functions is significantly higher in China than that in other regions (33). Furthermore, a recent cross-regional comparative study conducted by researchers from China and Pakistan has further confirmed that the distribution of virulence genes in *S. agalactiae* is closely associated with antibiotic resistance patterns and local farming practices (71, 172). In contrast, in regions with extensive management or restricted antimicrobial use, pathogens may rely more heavily on adhesion factors (33, 173). A study conducted in Iran reported a high prevalence of the *ebpS* and *bbp* genes, which were not detected in studies from other countries (30). Additionally, different hygiene conditions can affect pathogen selection (174). Meanwhile, differences in the genetic background of local dairy cow breeds across countries contribute to variations in disease resistance (175). The combined effects of these multiple factors may underlie the observed geographical disparities. The reason why different pathogens induce distinct clinical outcomes (CM and SCM) lies in their employment of distinct pathogenic mechanisms, they initially colonize the mammary gland via adhesion, and ultimately establish acute inflammation and chronic infection through factors with tissue-damaging or immune evasion functions. Notably, due to the unique characteristics of mammary gland tissue, iron acquisition is crucial for the proliferation of pathogenic bacteria. For instance, strains lacking the Fec System fail to induce mastitis, whereas the acquisition of Fec System enables other non-pathogenic strains to successfully infect the mammary gland (103, 115).

Regarding vaccine design targeting virulence factors, conserved and functionally indispensable VFs play pivotal roles in pathogen colonization and infection in the host, which justifies their identification as vaccine targets. However, their excellent antigenic conservation must also be considered. For instance, the prevalence of the *clfA* gene of *S. aureus*s is in general high across multiple countries and has been validated as an effective vaccine target in animal models. Furthermore, IsdB is highly expressed under iron-deficient conditions, functioning as a siderophore for nutrient acquisition with conserved biological activity, making it an ideal component for the development of multivalent vaccines (162). Similarly, FimH of *E. coli* serves as the primary mediator of bacterial colonization and is also a promising target for vaccine development (91). Future vaccine research and development should prioritize these core virulence factors and integrate multi-antigen strategies to enhance cross-protective efficacy.

## Conclusions and future perspectives

Bovine mastitis causes significant economic and production losses by reducing the milk production and milk quality of dairy cows, increasing culling rates, and raising treatment costs. The pathogenicity of the causal pathogens is influenced by host factors and the presence of an arsenal of VFs. Comprehensive studies have shown that the prevalence of these virulence genes varies across countries. Even within the same country, the prevalence of identical bacterial strains can differ, likely due to local environmental conditions and patterns of antimicrobial use. Regarding this point, more recent research needs to be combined for a comprehensive analysis. To better understand these variations, recent research must be integrated for more comprehensive analysis.

During infection, pathogens use VFs with adhesive functions to colonize the mammary gland, and subsequently damage host tissues through toxins and other virulence mechanisms. Notably, some of these VFs are relatively conserved and are commonly found in strains isolated from different regions. These conserved elements offer targets for therapeutic interventions.

To date, several therapeutic strategies, including vaccines, have been evaluated. However, no single method has proven universally effective, as pathogens respond differently to therapeutic techniques. Therefore, future research should prioritize understanding the interactions between pathogens and bovine hosts, with a focus on pathogen adaptability and host specificity. Additionally, longitudinal monitoring studies should be conducted to explore the evolution of virulence gene profiles in different farming systems and evaluate the impacts of environmental and management factors on the adaptive evolution of pathogens. Meanwhile, efforts can be intensified to investigate the synergistic effects of other emerging therapies such as bacteriophages combined with probiotics, vaccines paired with immunomodulators, and traditional Chinese medicine integrated with nanoformulations, so as to overcome the limitations of single therapies. Such insight will form the basis for the development of effective, region-specific control strategies aimed at the comprehensive prevention and control of bovine mastitis.

## Glossary

AMR: Antimicrobial resistance
ATP: Adenosine triphosphate
BMECs: Bovine mammary epithelial cells
CM: Clinical mastitis
CP: Capsular polysaccharide
ClfA: Clumping factor A
*E. coli*: *Escherichia coli*
ESBL: Extended-spectrum *β*-lactamase
Fn: Fibronectin
Fec: Ferric citrate
FnBPA: Fibronectin-binding proteins A
IMI: Intramammary infection
*K. pneumoniae*: *Klebsiella pneumoniae*
LPF: Long polar fimbriae
LPS: Lipopolysaccharide
LTA: Lipoteichoic Acid
*M. bovis*: *Mycoplasma bovis*
MSCRAMMs: Microbial surface components recognizing adhesive matrix molecules
MRSA: Methicillin-resistant *Staphylococcus aureus*
NETs: Neutrophil extracellular traps
NF-κB: Nuclear factor-kappa B
*S. aureus*: *Staphylococcus aureus*
*S. agalactiae*: *Streptococcus agalactiae*
Stx: Shiga toxins
SCM: Subclinical mastitis
*S. dysgalactiae*: *Streptococcus dysgalactiae*
TRAP: Thrombin-sensitive protein
VF: Virulence factor
Vsps: Variable surface lipoproteins
