# Supplementary material for: Virulence factors of bovine mastitis pathogens: distribution, pathogenesis, and emerging vaccines targeting virulence factors: a literature review
Source: Front Vet Sci. 2026 Jan 28;12:1745390. doi: 10.3389/fvets.2025.1745390 (PMC12892978; doi:10.3389/fvets.2025.1745390)
Supplement: Supplementary file 1 [file Table_1.docx]

**Methods for Selection of Literature**

This review was conducted by searching the PubMed and Web of Science databases. The specific search strategy used is as follows (taking PubMed as an example): ("bovine mastitis"[All Fields]) AND ("virulence"[All Fields] OR "prevalence"[All Fields] OR "pathogen"[All Fields] OR "mechanism"[All Fields] OR "treatment"[All Fields])，Inclusion criteria: studies published between 2014 and 2025, focusing on virulence genes, prevalence, pathogenic mechanisms, or vaccines of bovine mastitis pathogens; language restricted to English. Exclusion criteria: non-original studies (e.g., reviews, commentaries), studies involving non-bovine species, and studies with incomplete data or unavailable full texts. Data on gene prevalence were extracted from each study, and statistical analysis was performed using chi-square test in SPSS 26.0, with a significance level set at *p* < 0.05.
