# Supplementary material for: Virulence factors of bovine mastitis pathogens: distribution, pathogenesis, and emerging vaccines targeting virulence factors: a literature review
Source: Front Vet Sci. 2026 Jan 28;12:1745390. doi: 10.3389/fvets.2025.1745390 (PMC12892978; doi:10.3389/fvets.2025.1745390)
Supplement: Supplementary file 2 [file Table_2.docx]

Attachment: The distribution of virulence genes in bovine mastitis pathogens

CM: Clinical Mastitis; SCM: Subclinical Mastitis

| **Virulence genes** | **Source of *Staphylococcus aureus*** | | | | | | | | | | | | |
| --- | --- | --- | --- | --- | --- | --- | --- | --- | --- | --- | --- | --- | --- |
|  | Ethiopia [N (%)] | China [N (%)] | China [N (%)] | India [N (%)] | Brazil [N (%)] | China [N (%)] | Thailand [N (%)] | China [N (%)] | India [N (%)] | Argentina, Brazil, Germany, Italy, USA, South Africa [N (%)] | South Africa [N (%)] | USA [N (%)] | Iran [N (%)] |
|  | N = 68 | N = 28 | N = 298 | N = 42 | N = 57 | N = 35 | N =24 | N = 39 | N = 55 | N = 93 | N = 38 | N = 138 | N = 75 |
| *tst-1* |  |  |  |  |  | 14 (40) |  |  |  |  |  |  |  |
| *eta* | 17 (25) |  | 4 (1) |  |  |  |  |  |  |  |  |  |  |
| *clfA* | 15 (22) | 25 (89) | 292 (98) |  | 46 (81) | 27 (77) | 24 (100) |  |  |  |  |  | 63 (84) |
| *clfB* |  | 24 (86) | 288 (97) |  | 42 (74) |  |  |  |  |  |  |  | 63 (84) |
| *icaA* |  |  |  |  | 38 (67) |  |  |  |  |  |  |  |  |
| *icaD* |  |  |  |  | 54 (95) |  |  |  |  |  |  |  |  |
| *coo* |  | 28 (100) |  |  |  |  |  |  |  |  |  |  |  |
| *Ig* |  | 28 (100) |  |  |  |  |  |  |  |  |  |  |  |
| *eno* |  | 28 (100) |  |  |  |  |  |  |  |  |  |  |  |
| *hla* |  | 27 (96) |  |  |  | 33 (94) | 23 (96) | 33 (85) | 27 (49) | 93 (100) | 19 (50) |  |  |
| *hlb* |  | 26 (93) |  |  |  | 34 (97) |  | 32 (82) | 33 (60) | 79 (85) | 19 (50) | 13 (9) |  |
| *nuc* |  | 24 (86) |  | 42 (100) |  | 30 (86) |  | 35 (90) |  |  |  |  |  |
| *pvl* |  |  |  | 20 (48) |  |  | 23 (96) |  |  |  |  |  |  |
| *spa* |  |  |  | 39 (93) |  |  | 24 (100) |  |  |  | 20 (53) |  | 74 (99) |
| *fib* |  |  |  |  | 53 (93) |  |  |  |  |  |  |  |  |
| *fnbA* |  |  | 288 (97) |  | 47 (83) |  |  | 38 (97) |  |  |  |  |  |
| *bap* |  |  |  |  | 45 (79) |  |  |  |  |  | 1 (3) |  |  |
| *sea* |  |  | 52 (17) |  |  |  |  |  |  | 61 (66) | 5 (13) |  |  |
| *see* |  |  |  |  | 37 (65) |  |  |  |  |  |  |  |  |
| *sec* |  |  | 22 (7) |  |  |  | 19 (79) |  |  |  |  |  |  |
| *sed* |  |  | 5 (2) |  | 35 (61) |  |  |  |  |  |  |  |  |
| *coa* |  |  |  | 42 (100) |  | 33 (94) | 24 (100) |  | 49 (89) |  | 21 (55) |  |  |
| *fnbpA* |  |  |  |  |  | 33 (94) |  |  |  |  |  |  |  |
| *fnbpB* |  |  |  |  |  | 28 (80) |  |  |  |  |  |  |  |
| *blaZ* |  |  |  |  |  |  |  |  | 51 (93) |  |  |  |  |
| *aur* |  |  |  |  |  |  |  |  |  |  |  | 138 (100) |  |
| *hlg* |  |  |  | 34 (81) |  |  |  |  |  |  |  |  |  |
| *hlgA* |  |  |  |  |  |  |  |  |  |  |  | 137 (99) |  |
| *hlgB* |  |  |  |  |  |  |  |  |  |  |  | 138 (100) |  |
| *hlgC* |  |  |  |  |  |  |  |  |  |  |  | 137 (99) |  |
| *ebpS* |  |  | 109 (37) |  |  |  |  |  |  |  |  |  | 73 (97) |
| *fnb* |  |  |  |  |  |  |  |  |  |  |  |  | 73 (97) |
| *bbp* |  |  |  |  |  |  |  |  |  |  |  |  | 65 (87) |
| *cna* |  |  |  |  |  |  |  |  |  |  |  |  | 49 (65) |
| Sample sources | SCM | SCM | CM and SCM | CM and SCM | SCM | CM (n=5), SCM (n=30) | SCM | CM | CM | CM | SCM | Packaged milk | CM and SCM |
| References | Tegegne et al., 2021 [36] | Xu et al., 2015 [34] | Zhang et al., 2022 [33] | Roshan et al., 2022 [42] | Nuñez et al., 2023 [32] | Wang et al., 2016 [35] | Pumipuntu et al., 2019 [30] | Yang et al., 2012 [37] | Neelam et al., 2022 [39] | Monistero et al., 2020 [38] | Khasapane et al., 2024 [40] | Patel et al., 2021 [41] | Ahangari et al., 2017 [31] |

| **Virulence genes** | **Source of *Streptococcus agalactiae*** | | | | | | | | |  |  |
| --- | --- | --- | --- | --- | --- | --- | --- | --- | --- | --- | --- |
|  | **India [N**  **(%)]** | **Egypt [N**  **(%)]** | **China [N**  **(%)]** | **Argentina [N**  **(%)]** | **China [N**  **(%)]** | **Colombia [N**  **(%)]** | **China[N**  **(%)]** | **Poland [N (%)]** | **China [N**  **(%)]** | **China [N**  **(%)]** | **Pakistan [N**  **(%)]** |
|  | **N = 42** | **N = 22** | **N = 57** | **N = 56** | **N = 140** | **N = 181** | **N = 324** | **N = 68** | **N = 105** | **N = 104** | **N = 166** |
| *scpB* | 15 (36) |  | 4 (5) |  | 1 (1) | 73 (40) | 274 (85) | 24 (35) |  |  |  |
| *cfb* | 16 (38) |  | 32 (56) |  | 139 (99) | 180 (100) |  | 68 (100) | 105 (100) | 104 (100) | 166 (100) |
| *cycle* | 10 (24) | 15 (68) |  | 56 (100) |  |  |  |  |  |  |  |
| *hylB* |  | 22 (100) | 28 (49) | 56 (100) | 139 (99) | 181 (100) | 320 (99) | 68 (100) | 105 (100) | 104 (100) | 166 (100) |
| *loop* |  |  |  |  |  | 181 (100) |  |  |  |  |  |
| *bca* |  |  | 3 (5) | 20 (36) | 3 (2) |  |  | 6 (9) |  | 24 (23) | 40 (24) |
| *iagA* |  | 3 (14) |  |  |  |  |  |  |  |  |  |
| *cylE* |  | 15 (68) |  |  | 136 (97) |  |  | 65 (96) | 105 (100) | 104 (100) | 166 (100) |
| *fbsA* |  |  |  |  | 139 (99) |  |  | 63 (93) | 105 (100) |  |  |
| *fbsB* |  |  |  |  |  |  |  |  |  | 104 (100) | 166 (100) |
| *bac* |  | 22 (100) |  |  |  |  |  | 11 (16) |  |  | 13 (8) |
| *cyl* |  |  | 39 (68) |  |  |  | 324 (100) |  |  |  |  |
| *glnA* |  |  | 27 (47) |  |  |  | 248 (77) |  |  |  |  |
| *scaA* |  |  | 26 (46) |  |  |  | 224 (69) |  |  |  |  |
| *lmb* |  |  | 17 (30) |  | 22 (16) |  |  |  |  |  |  |
| *cjb* |  |  |  |  |  |  | 320 (99) |  |  |  |  |
| *spb1* |  |  |  | 56 (100) |  |  |  |  |  |  |  |
| *PI-2b* |  |  |  | 56 (100) |  |  |  |  |  |  |  |
| *cpsA* |  |  |  | 54 (96) |  |  |  |  |  |  |  |
| *rib* |  |  |  | 33 (59) |  |  |  | 7 (10) |  |  |  |
| *lacIV* |  |  |  |  | 140 (100) |  |  |  |  |  |  |
| *gapC* |  |  |  |  | 140 (100) |  |  |  |  |  |  |
| *dltA* |  |  |  |  | 140 (100) |  |  |  |  |  |  |
| *lacIII* |  |  |  |  | 139 (99) |  |  |  |  |  |  |
| *lacI* |  |  |  |  | 138 (99) |  |  |  |  |  |  |
| *bibA* |  |  |  |  | 137 (98) |  |  |  |  | 48 (46) | 51 (31) |
| *lacII* |  |  |  |  | 129 (92) |  |  |  |  |  |  |
| *cspA* |  |  |  |  | 73 (52) |  |  | 68 (100) |  | 19 (18) |  |
| *pavA* |  |  |  |  | 35 (25) |  |  |  |  |  |  |
| *sip* |  |  |  |  |  |  |  | 68 (100) |  |  |  |
| Sample sources | CM and SCM | SCM | CM | CM and SCM | CM | CM and SCM | CM | CM and SCM | CM | CM and SCM | CM and SCM |
| References | Parasana et al., 2022 [69] | Abd El-Razik et al., 2021 [74] | Ding et al., 2016 [66] | Hernandez et al., 2021 [75] | Liu et al., 2024 [67] | Torres et al., 2023 [71] | Zhang et al., 2019 [68] | Zastempowska et al., 2022 [70] | Han et al., 2022 [72] | [Leghari](https://webofscience.clarivate.cn/wos/author/record/10830235) et al., 2023 [73] | [Leghari](https://webofscience.clarivate.cn/wos/author/record/10830235) et al., 2023 [73] |

| **Virulence genes** | **Source of *Mycoplasma bovis*** | |
| --- | --- | --- |
|  | Australia [N (%)] | Iran [N (%)] |
|  | N = 82 | N = 21 |
| *clpC* | 78 (95) |  |
| *tufa* | 81 (99) |  |
| *MAG006* | 64 (78) |  |
| *LppB* |  | 11 (52) |
| *P48* |  | 12 (57) |
| Sample sources | Mastitis and healthy cows | CM and SCM |
| References | Parker et al., 2016 [82] | Ghazvineh et al., 2024 [225] |

| **virulence genes** | **Source of *Escherichia coli*** | | | | | | | | | | | | | | |
| --- | --- | --- | --- | --- | --- | --- | --- | --- | --- | --- | --- | --- | --- | --- | --- |
|  | Brazil [N (%)] | Switzerland [N (%)] | China [N (%)] | Egypt [N (%)] | China [N (%)] | Pakistan [N (%)] | Iran [N (%)] | Iran [N (%)] | Brazil [N (%)] | Iran [N (%)] | Turkey [N (%)] | Ireland [N (%)] | Vietnam [N (%)] | South Korea [N (%)] | Jordan [N (%)] |
|  | N = 110 | N = 82 | N = 79 | N = 15 | N = 87 | N = 30 | N = 70 | N = 42 | N = 114 | N = 47 | N = 155 | N = 37 | N = 50 | N = 183 | N = 14 |
| *Aer* |  |  |  |  | 87 (100) |  |  |  |  |  |  |  |  |  |  |
| *Sfa* |  |  |  |  |  |  |  |  |  |  |  |  |  | 44 (24) |  |
| *stx1* |  |  |  |  |  |  |  |  |  | 34 (72) |  |  |  |  |  |
| *eaeA* |  |  | 1 (1) |  |  |  | 2 (3) |  |  | 42 (89) |  |  |  |  |  |
| *traT* | 85 (77) | 59 (72) |  |  | 40 (46) | 8(27) |  |  | 93 (82) |  | 103 (67) |  |  | 49 (27) |  |
| *afaD-8* |  |  |  |  |  |  | 5 (7) | 4(10) |  |  |  |  |  |  |  |
| *afaE-8* |  |  |  |  |  |  | 3(4) | 4(10) |  |  |  |  |  |  |  |
| *iucD* | 4 (4) |  | 1 (1) |  | 9 (10) | 9(30) | 3 (4) |  |  |  |  |  |  |  |  |
| *irp2* | 38 (35) |  | 2 (3) |  | 11 (13) |  |  |  | 11 (10) |  |  |  |  |  |  |
| *iss* |  |  |  |  |  |  |  |  |  |  |  | 15 (41) |  | 70 (38) |  |
| *astA* |  |  |  |  | 9 (10) |  |  |  |  |  |  | 5 (14) |  |  |  |
| *tsh* |  |  |  | 9 (60) |  |  |  |  |  |  |  |  |  |  |  |
| *vat* | 1 (1) | 3 (4) |  |  |  |  |  |  |  |  |  |  |  |  |  |
| *f17c-A* |  |  |  |  |  |  |  | 6 (14) |  |  |  |  |  |  |  |
| *eae* |  |  |  |  |  |  |  | 6 (14) |  |  |  |  | 2 (4) |  |  |
| *f17b-A* |  |  |  |  |  |  |  | 5 (12) |  |  |  |  |  |  |  |
| *f17d-A* |  |  |  |  |  |  |  | 5 (12) |  |  |  |  |  |  |  |
| *aucD* |  |  |  |  |  |  |  | 4 (10) |  |  |  |  |  |  |  |
| *bfpA* |  |  |  |  |  |  |  | 4 (10) |  |  |  |  |  |  |  |
| *clpG* |  |  |  |  |  |  |  | 2 (5) |  |  |  |  |  |  |  |
| *VT* |  |  |  |  |  |  |  | 2 (5) |  |  |  |  |  |  |  |
| *fimH* | 103 (94) |  | 71 (90) | 12 (80) |  |  |  |  | 114 (100) |  |  |  |  | 148 (81) |  |
| *ecpA* | 72 (65) |  |  |  |  |  |  |  | 73 (64) |  |  |  |  |  |  |
| *fimA* | 31 (28) |  |  |  |  |  |  |  | 36 (32) |  |  |  |  |  |  |
| *ompT* | 75 (68) |  |  |  |  |  |  |  | 40 (35) |  |  |  |  | 47 (26) |  |
| *hlyA* |  | 15 (18) |  |  | 7 (8) |  |  |  | 8 (7) | 9 (19) |  |  |  |  |  |
| *F41* |  |  |  |  |  |  |  |  |  | 1 (2) |  |  |  |  |  |
| *sta* |  |  |  |  |  |  |  |  |  | 4 (9) |  |  |  |  |  |
| *lpfA* |  |  |  |  |  |  |  |  |  |  |  | 7 (19) |  |  |  |
| *tetA* |  |  |  | 15 (100) |  |  |  |  |  |  |  |  | 6 (12) |  | 13 (93) |
| *tetB* |  |  |  |  |  |  |  |  |  |  |  |  | 6 (12) |  | 14 (100) |
| *sul1* |  |  |  | 15 (100) |  |  |  |  |  |  |  |  | 13 (26) |  | 11 (79) |
| *sul2* |  |  |  |  |  |  |  |  |  |  |  |  | 15 (30) |  |  |
| *iutA* |  | 17 (21) |  |  |  |  |  |  |  |  |  |  | 1 (2) |  |  |
| *stx2* |  |  |  |  |  |  |  |  |  |  |  |  | 3 (6) |  |  |
| *fyuA* |  | 23 (28) |  |  |  |  |  |  |  |  |  |  |  |  |  |
| *papA* | 1 (1) |  |  |  |  |  |  |  |  |  |  |  |  |  |  |
| *ereA* |  |  |  |  |  |  |  |  |  |  |  |  |  |  | 14 (100) |
| *tetG* |  |  |  |  |  |  |  |  |  |  |  |  |  |  | 14 (100) |
| *tetE* |  |  |  |  |  |  |  |  |  |  |  |  |  |  | 14 (100) |
| *ampC* |  |  |  |  |  |  |  |  |  |  |  |  |  |  | 12 (86) |
| *strA* |  |  |  |  |  |  |  |  |  |  |  |  |  |  | 12 (86) |
| *tetD* |  |  |  |  |  |  |  |  |  |  |  |  |  |  | 10 (71) |
| *tetC* |  |  |  |  |  |  |  |  |  |  |  |  |  |  | 8 (57) |
| *aadA* |  |  |  |  |  |  |  |  |  |  |  |  |  |  | 8 (57) |
| *strB* |  |  |  |  |  |  |  |  |  |  |  |  |  |  | 5 (36) |
| *bla1* |  |  |  |  |  |  |  |  |  |  |  |  |  |  | 3 (21) |
| *bla2* |  |  |  |  |  |  |  |  |  |  |  |  |  |  | 2 (14) |
| *iroN* | 4 (4) |  |  |  |  |  |  |  |  |  |  |  |  |  |  |
| *sitA* | 29 (26) |  |  |  |  |  |  |  |  |  |  |  |  |  |  |
| *ompC* |  |  | 79 (100) |  |  |  |  |  |  |  |  |  |  |  |  |
| *ompF* |  |  | 58 (73) |  |  |  |  |  |  |  |  |  |  |  |  |
| *ompA* |  |  |  |  | 87 (100) |  |  |  |  |  |  |  |  |  |  |
| Sample sources | CM | CM and SCM | CM | SCM | CM and SCM | CM and SCM | SCM | CM | CM | CM | CM | CM | CM | Packaged milk | CM |
| References | Campos et al., 2022 [96] | Nüesch-In et al., 2019 [95] | Zhang et al., 2018 [97] | Abed et al., 2021 [99] | Zuo et al., 2025 [92] | Aslam et al., 2021 [101] | Marashifard et al., 2019 [102] | Jamali et al., 2018 [221] | Guerra et al., 2019 [93] | Aflakian et al., 2022 [94] | Günaydin et al., 2016 [100] | Keane et al., 2016 [223] | My et al., 2023 [201] | Kang et al., 2022 [98] | Ismail et al., 2020 [103] |

| **Virulence genes** | **Source of *Streptococcus uberis*** | | | | | |
| --- | --- | --- | --- | --- | --- | --- |
|  | Czech Republic [N (%)] | Egypt [N (%)] | China [N (%)] | Brazil [N (%)] | Thailand [N (%)] | Argentina [N (%)] |
|  | N = 190 | N = 69 | N = 16 | N = 46 | N = 88 | N = 78 |
| *hasA* | 179 (94) |  | 2 (13) |  | 40 (45) | 58 (74） |
| *hasB* | 179 (94) |  | 3 (19) |  | 40 (45) | 52 (67） |
| *hasC* | 118 (62) |  | 14 (88) |  | 63 (72) | 70 (90） |
| *sua* | 190 (100) | 29 (42) | 13 (81) | 46 (100) | 62 (71) | 65 (83） |
| *cfu* | 11 (6) | 15 (22) | 9 (56) |  | 25 (28) | 60 (77） |
| *lbp* | 4 (2) |  | 4 (25) |  | 55 (62) | 9 (12） |
| *skc* | 186 (98) | 14 (20) |  | 42 (91) |  | 51 (65） |
| *pauA* | 184 (97) | 27 (39) | 13 (81) | 42 (91) | 52 (59) | 48 (62） |
| *gapC* | 190 (100) |  | 16 (100) |  | 63 (72) | 62 (79） |
| *oppF* | 190 (100) |  |  |  | 64 (73) | 50 (64） |
| *opp* |  | 8 (12) |  |  |  |  |
| *mtuA* |  |  |  |  | 62 (71) |  |
| Sample sources | CM and SCM | CM | CM | CM and SCM | CM and SCM | CM and SCM |
| References | Zouharova et al., 2022 [121] | Abd El-Aziz et al., 2021 [122] | Zhang et al., 2020 [123] | Loures et al., 2017[124] | Boonyayatra et al., 2018[125] | Reinoso et al., 2011[126] |

| **Virulence genes** | **Source of *Klebsiella pneumoniae*** | | | | | | |
| --- | --- | --- | --- | --- | --- | --- | --- |
|  | India [N (%)] | China [N (%)] | China [N (%)] | China [N (%)] | Egypt [N (%)] | USA [N (%)] | China [N (%)] |
|  | N = 27 | N = 431 | N = 124 | N = 68 | N = 35 | N = 180 | N = 239 |
| *wabG* | 25 (93) |  |  | 68 (100) |  |  | 234 (98) |
| *kfuBC* | 5 (19) |  |  | 34 (50) |  |  |  |
| *entB* |  | 399 (93) | 97 (78) |  |  | 37 (21) | 232 (97) |
| *kfu* |  | 271 (63) | 38 (31) |  | 28 (80) |  | 85 (36) |
| *rpmA* |  | 19 (4) |  | 3 (4) | 28 (80) |  |  |
| *fimH1* |  | 384 (89) | 68 (55) | 68 (100) |  |  |  |
| *mrkD* |  | 425 (99) | 30 (24) |  |  |  | 236 (99) |
| *uge* |  |  |  | 68 (100) | 28 (80) |  | 209 (87) |
| *β-d-lacZ* |  | 416 (97) |  |  |  |  |  |
| *iutA* |  | 40 (9) |  |  |  |  | 236 (99) |
| *Any* |  | 428 (99) |  |  |  |  |  |
| *nif* |  | 19 (4) |  |  |  |  |  |
| *allS* |  | 57 (13) |  |  |  |  | 21 (9) |
| *ureA* |  |  |  | 68 (100) |  |  | 237 (99) |
| *wcaG* |  |  |  | 8 (12) |  |  | 2 (1) |
| *ybtA* |  |  |  | 16 (24) |  |  | 10 (4) |
| *iucB* |  |  |  | 36 (53) |  |  |  |
| *magA* |  |  |  |  | 28 (80) |  | 1 (0) |
| *kfuABC* |  |  |  |  |  | 99 (55) |  |
| *terB* |  |  |  |  |  |  | 32 (13) |
| *iucA* |  |  |  |  |  |  | 3 (1) |
| *irp2* |  |  |  |  |  |  | 14 (6) |
| *iroN* |  |  |  |  |  |  | 225 (94) |
| *fimH* |  |  |  |  |  |  | 230 (96) |
| Sample sources | Raw milk (n=17), mastitis (n=10) | CM (n=129), SCM (n=77), bulk tank milk (n=66), environment and extramammary sites (n=159) | CM | CM | CM | CM and SCM | Mastitis and healthy cows |
| References | Katira et al., 2024 [224] | Cheng et al., 2021 [137] | Gao et al., 2019 [138] | Xu et al., 2022[142] | Osman et al., 2014 [141] | Zheng et al., 2022 [140] | Wu et al., 2022 [139] |

| **virulence genes** | **Source of *Streptococcus dysgalactiae*** | | | | |
| --- | --- | --- | --- | --- | --- |
|  | America[N (%)] | China[N (%)] | Portugal[N (%)] | China[N (%)] | China[N (%)] |
|  | N = 35 | N = 60 | N = 37 | N = 13 | N = 10 |
| *perR* | 35 (100) |  |  |  |  |
| *leus* | 33 (94) |  |  |  |  |
| *gldA* | 32 (91) |  |  |  |  |
| *hasc* | 32 (91) |  |  |  |  |
| *scpB* |  | 11 (18) |  | 2 (15) | 2 (20) |
| *bca* |  | 4 (7) |  | 2 (15) | 2 (20) |
| *purH* | 32 (91) |  |  |  |  |
| *lmb* | 25 (66) | 2 (3) |  | 3 (15) | 6 (60) |
| *SP_0251* | 32 (91) |  |  |  |  |
| *SpyM3_0013* | 32 (91) |  |  |  |  |
| *cydA* | 32 (91) |  |  |  |  |
| *mf/spd* | 31 (89) |  |  |  |  |
| *rpOE* | 31 (89) |  |  |  |  |
| *SP_0I21* | 31 (89) |  |  |  |  |
| *SP 0494* | 31 (89) |  |  |  |  |
| *oppA* | 31 (89) |  |  |  |  |
| *gInA* | 30 (86) |  |  |  |  |
| *fba* | 30 (86) |  |  |  |  |
| *luxS* | 30 (86) |  |  |  |  |
| *covS* | 30 (86) |  |  |  |  |
| *atmB* | 30 (86) |  |  |  |  |
| *clpP* | 29 (83) |  |  |  |  |
| *PurB* | 29 (83) |  |  |  |  |
| *SPy_1718* | 29 (83) |  |  |  |  |
| *vicK* | 29 (83) |  |  |  |  |
| *ccpA* | 29 (83) |  |  |  |  |
| *SPy_1633* | 28 (80) |  |  |  |  |
| *fbp54* | 28 (80) |  |  |  |  |
| *SP_2086* | 28 (80) |  |  |  |  |
| *SP_1970* | 27 (77) |  |  |  |  |
| *SP_0095* | 27 (77) |  |  |  |  |
| *dItA* | 27 (77) |  |  |  |  |
| *lgt* | 26 (74) |  |  |  |  |
| *lsp* | 25 (71) |  |  |  |  |
| *SP_0320* | 25 (71) |  |  |  |  |
| *SP_1396* | 25 (71) |  |  |  |  |
| *SP_1398* | 25 (71) |  |  |  |  |
| *cpsY* | 24 (69) |  |  |  |  |
| *lepA* | 24 (69) |  |  |  |  |
| *SP_0829* | 24 (69) |  |  |  |  |
| *SP_0856* | 24 (69) |  |  |  |  |
| *EF1623* | 23 (66) |  |  |  |  |
| *guaA* | 22 (63) |  |  |  |  |
| *mf3* | 17 (49) |  |  |  |  |
| *sda* | 8 (23) |  |  |  |  |
| *emm* | 6 (17) |  |  |  |  |
| *emm1* | 6 (17) |  |  |  |  |
| *SpyM3_0386* | 6 (17) |  |  |  |  |
| *speK* | 4 (11) |  | 15 (41) |  |  |
| *SP_0338* | 2 (6) |  |  |  |  |
| *SP_1399* | 2 (6) |  |  |  |  |
| *napr* |  | 60 (100) |  |  |  |
| *cfb* |  | 22 (37) |  | 4 (31) |  |
| *eno* |  | 10 (17) |  |  |  |
| *cyl* |  | 10 (17) |  | 2 (15) |  |
| *speC* |  |  | 7 (19 ) |  |  |
| *speL* |  |  | 7 (19) |  |  |
| *speM* |  |  | 9 (24) |  |  |
| *spd1* |  |  | 7 (19) |  |  |
| *sdn* |  |  | 10 (27) |  |  |
| *sagA* |  |  | 37 (100) |  |  |
| *bac* |  |  |  | 2 (15) |  |
| *hylB* |  |  |  | 2 (15) | 4 (40) |
| Sample source | CM | CM | CM and SCM | CM and SCM | CM and SCM |
| References | Crippa et al., 2023 [155] | Shen et al., 2021 [152] | Alves-Barroco et al., 2021 [156] | Tian et al., 2019 [153] | Dhital et al., 2023 [154] |
