# Supplementary material for: Virulence factors of bovine mastitis pathogens: distribution, pathogenesis, and emerging vaccines targeting virulence factors: a literature review
Source: Front Vet Sci. 2026 Jan 28;12:1745390. doi: 10.3389/fvets.2025.1745390 (PMC12892978; doi:10.3389/fvets.2025.1745390)
Supplement: Supplementary file 3 [file Table_3.docx]

| *Staphylococcus aureus* | **Antibiotics** | | **Resistance rate** | | | | | | | | | | | | | | **Average resistance rate** |
| --- | --- | --- | --- | --- | --- | --- | --- | --- | --- | --- | --- | --- | --- | --- | --- | --- | --- |
|  | Beta-lactam | Imipenem | 16.4% |  |  |  |  |  |  |  |  |  |  |  |  |  | 16.4% |
|  |  | Ceftiofur |  | 31.0% |  |  |  |  |  | 2.1% |  |  |  |  |  |  | 16.5% |
|  |  | Ampicillin |  |  |  |  |  |  |  | 65.0% |  |  |  |  | 4.9% |  | 34.9% |
|  |  | Oxacillin |  |  |  |  |  | 55.9% |  | 3.1% |  | 25.0% |  |  |  |  | 28.0% |
|  |  | Penicillin |  | 60.7% | 58.7% | 100.0% | 46.0% | 11.0% | 92.0% | 89.7% | 58.5% |  | 75.7% | 10.8% | 87.8% | 30.0% | 60.1% |
|  |  | Amoxycillin |  |  |  |  |  | 42.0% |  |  |  |  |  |  |  |  | 42.0% |
|  |  | Ceftriaxone | 79.1% |  |  |  |  | 0.0% |  |  |  |  |  |  |  |  | 39.6% |
|  |  | Meropenem | 100.0% |  |  |  |  |  |  |  |  |  |  |  |  |  | 100.0% |
|  |  | Cephalexin |  |  |  |  |  |  |  | 1.0% |  |  |  | 10.8% |  |  | 5.9% |
|  |  | Cefotaxime | 40.3% |  |  |  |  |  |  |  |  |  |  |  |  | 0.0% | 20.1% |
|  |  | Ceftazidime |  |  |  |  |  |  |  |  |  |  |  |  | 0.0% |  | 0.0% |
|  |  | Cefoxitin |  |  |  |  |  |  | 25.8% |  |  | 25.0% |  |  |  |  | 25.4% |
|  | Aminoglycosides | Gentamicin | 71.6% |  | 10.5% | 0.0% | 0.0% | 17.9% | 53.9% |  | 10.8% | 58.8% | 1.4% |  | 17.1% |  | 24.2% |
|  |  | Streptomycin |  |  |  | 16.5% |  |  |  |  |  |  | 27.1% | 44.6% | 100.0% |  | 47.1% |
|  |  | Amikacin | 100.0% |  |  |  |  |  |  |  |  |  |  |  |  |  | 100.0% |
|  |  | Kanamycin |  |  |  |  |  |  | 65.1% |  |  |  |  |  | 7.3% |  | 36.2% |
|  |  | Neomycin |  |  |  | 45.8% |  |  |  |  |  |  |  | 21.5% | 0.0% |  | 22.4% |
|  | Fluoroquinolones | Norfloxacin |  |  |  |  |  |  |  |  | 4.6% |  |  |  | 0.0% |  | 2.3% |
|  |  | Nalidixic acid | 31.3% |  |  |  |  |  |  |  |  |  |  |  |  |  | 31.3% |
|  |  | Enrofloxacin |  |  |  |  |  |  |  |  |  |  |  |  |  | 6.7% | 6.7% |
|  |  | Ciprofloxacin | 100.0% |  | 8.7% |  | 0.0% | 49.6% | 49.0% |  | 4.6% |  |  |  | 0.0% |  | 30.3% |
|  | Tetracyclines | Oxytetracycline |  |  |  | 8.3% |  | 74.5% |  |  |  |  | 48.6% |  |  |  | 43.8% |
|  |  | Tetracycline | 34.3% | 48.8% | 15.1% | 83.3% | 26.0% |  | 24.5% |  | 12.3% | 30.0% |  | 10.8% | 4.9% |  | 29.0% |
|  |  | Doxycycline |  |  |  |  |  |  |  |  |  |  |  |  | 0.0% |  | 0.0% |
|  | Macrolides | Erythromycin |  | 54.8% | 22.1% |  | 18.0% | 8.2% |  |  |  | 40.0% |  |  | 85.4% |  | 38.1% |
|  |  | Azithromycin | 7.5% |  |  |  |  | 0.0% |  |  |  |  |  |  |  |  | 3.7% |
|  | Lincosamide | Lincomycin |  |  |  |  |  |  |  | 24.7% |  |  |  | 10.8% |  |  | 17.8% |
|  |  | Clindamycin |  |  |  |  |  |  | 56.0% |  | 40.0% | 53.8% | 4.3% |  |  |  | 38.5% |
|  | Sulfonamides | Sulfamethoxazole/Trimethoprim | 100.0% |  | 5.8% |  | 0.0% | 30.0% | 0.0% |  |  |  | 12.9% |  |  |  | 24.8% |
|  |  | Trimethoprim |  |  |  | 8.3% |  |  |  |  |  |  |  |  |  |  | 8.3% |
|  | Amide alcohol | Chloramphenicol | 46.3% |  |  |  |  |  | 8.7% |  | 1.5% |  |  |  |  |  | 18.8% |
|  | Polymyxins | Colistin sulfate | 4.5% |  |  |  |  |  |  |  |  |  |  |  |  |  | 4.5% |
|  | Sample size | | 67 | 84 | 172 | 24 | 39 | 145 | 298 | 97 | 65 | 80 | 70 | 65 | 41 | 30 |  |
|  | Countrys | | Bangladesh | Brazil | China | Iran | Malaysia | Bangladesh | China | China | China | India | Ethiopia | Slovakia | China | Turkiye |  |
|  | References | | Emon et al., 2024 [182] | Freu et al., 2022 [186] | Yang et al., 2023 [187] | Hemati et al., 2023 [188] | Saeed et al., 2022 [189] | Hoque et al., 2018 [190] | Zhang et al., 2022 [33] | Song et al., 2024 [191] | Ren et al., 2020 [192] | Brahma et al., 2022 [193] | Demil et al., 2022 [194] | Holko et al., 2019 [183] | Wang et al., 2024 [184] | Guner et al., 2024 [185] |  |

| *Streptococcus agalactiae* | **Antibiotics** | | **Resistance rate** | | | | | | | | | |  |  | **Average resistance rate** |
| --- | --- | --- | --- | --- | --- | --- | --- | --- | --- | --- | --- | --- | --- | --- | --- |
|  | Beta-lactam | Ceftiofur |  | 0.0% | 4.8% | 1.0% | 2.3% |  |  |  |  |  |  |  | 2.0% |
|  |  | Amoxycillin |  | 0.0% |  |  |  |  |  |  |  |  | 8.0% | 10.0% | 6.0% |
|  |  | Penicillin | 7.9% | 0.0% | 0.0% | 1.0% | 2.3% | 38.5% | 23.3% | 100.0% | 0.0% | 14.3% | 0.0% | 18.0% | 17.1% |
|  |  | Ampicillin | 36.8% |  | 28.6% | 0.0% | 1.7% |  |  | 0.0% |  |  | 3.0% | 17.0% | 12.4% |
|  |  | Oxacillin |  |  | 9.5% | 0.0% | 3.5% |  |  |  |  |  | 0.0% | 15.0% | 5.6% |
|  |  | Ceftazidime |  |  |  |  |  |  |  | 0.0% |  |  |  |  | 0.0% |
|  |  | Cephalexin |  |  |  |  |  |  | 10.0% |  |  |  |  |  | 10.0% |
|  |  | Cefotaxime |  |  |  |  |  |  |  |  |  | 14.3% |  |  | 14.3% |
|  | Aminoglycosides | Gentamicin |  |  | 4.8% |  |  | 11.5% |  | 92.1% | 100.0% | 14.3% | 0.0% | 3.0% | 32.2% |
|  |  | Kanamycin |  |  |  |  |  |  |  | 0.0% | 96.0% |  | 12.0% | 20.0% | 32.0% |
|  |  | Neomycin |  |  |  |  |  |  | 80.0% | 0.0% |  |  |  |  | 40.0% |
|  |  | Streptomycin |  |  |  |  |  | 61.5% | 70.0% | 100.0% |  |  |  |  | 77.2% |
|  | Fluoroquinolones | Enrofloxacin |  | 23.8% |  |  |  |  | 23.3% |  | 0.0% | 14.3% |  |  | 15.3% |
|  |  | Ciprofloxacin |  |  | 19.0% |  |  |  |  | 0.0% |  |  |  |  | 9.5% |
|  |  | Norfloxacin |  |  |  |  |  |  |  | 0.0% |  |  |  |  | 0.0% |
|  | Tetracyclines | Oxytetracycline |  |  |  |  |  | 76.9% |  |  |  |  |  |  | 76.9% |
|  |  | Tetracycline |  |  | 85.7% | 33.0% | 68.6% |  | 13.3% | 100.0% | 44.0% |  | 94.0% | 95.0% | 66.7% |
|  |  | Doxycycline |  | 71.4% | 33.3% |  |  |  |  | 0.0% |  |  |  |  | 34.9% |
|  | Macrolides | Erythromycin |  | 35.7% | 47.6% | 1.0% | 29.1% |  |  | 100.0% | 7.0% |  | 95.0% | 91.0% | 50.8% |
|  | Lincosamide | Clindamycin |  | 43.8% | 52.1% |  |  | 11.5% |  |  |  |  | 70.0% | 72.0% | 49.9% |
|  |  | Lincomycin | 44.7% | 42.9% |  |  |  |  | 6.7% |  |  |  | 1.0% | 5.0% | 20.06% |
|  | Sulfonamides | Sulfamethoxazole/Trimethoprim |  |  |  |  |  | 7.7% |  |  |  |  |  |  | 7.7% |
|  |  | Sulfamethoxazole |  |  | 4.8% |  |  |  |  |  |  |  |  |  | 4.8% |
|  | Amide alcohol | Chloramphenicol |  | 9.5% |  |  |  |  |  |  |  |  |  |  | 9.5% |
|  |  | Florfenicol |  | 11.9% | 4.8% |  |  |  |  |  |  |  |  |  | 8.4% |
|  | Rifamycins | Rifampicin |  | 2.4% |  |  |  |  |  |  |  |  | 21.0% | 27.0% | 16.8% |
|  | Sample size | | 38 | 42 | 21 | 100 | 86 | 26 | 30 | 76 | 27 | 7 | 100 | 100 |  |
|  | Countrys | | China | China | China | Thailand | Brazilian | Ethiopia | Slovakia | China | Poland | Turkiye | China | Pakistan |  |
|  | References | | Song et al., 2024 [191] | Lin et al., 2021 [195] | Ma et al., 2023 [196] | Wataradee et al., 2023 [4] | Tomazi et al., 2018 [197] | Demil et al., 2022 [194] | Holko et al., 2019 [183] | Wang et al., 2024 [184] | Kaczorek et al., 2017 [198] | Guner et al., 2024 [185] | [Leghari](https://webofscience.clarivate.cn/wos/author/record/10830235) et al., 2023 [73] | [Leghari](https://webofscience.clarivate.cn/wos/author/record/10830235) et al., 2023 [73] |  |

| *Mycoplasma bovis* | **Antibiotics** | | **Resistance rate** | | **Average resistance rate** |
| --- | --- | --- | --- | --- | --- |
|  | Aminoglycosides | Spectinomycin |  | 0.0% | 0.0% |
|  |  | Kanamycin | 100.0% |  | 100.0% |
|  | Fluoroquinolones | Danofloxacin | 0.0% |  | 0.0% |
|  |  | Enrofloxacin | 0.0% | 67.2% | 33.6% |
|  | Tetracyclines | Oxytetracycline | 100.0% |  | 100.0% |
|  |  | Doxycycline |  | 54.1% | 54.1% |
|  | Macrolides | Pirlimycin | 0.0% |  | 0.0% |
|  |  | Erythromycin |  | 0.0% | 0.0% |
|  |  | Tilmicosin | 100.0% | 0.0% | 50.0% |
|  |  | Tylosin | 100.0% | 0.0% | 50.0% |
|  | Sample size | | 30 | 61 |  |
|  | Countrys | | Japan | Egypt |  |
|  | References | | Kawai et al., 2014 [199] | Ammar et al., 2022 [200] |  |

| *Escherichia coli* | **Antibiotics** | | **Resistance rate** | | | | | | | | | | | | **Average resistance rate** |  |
| --- | --- | --- | --- | --- | --- | --- | --- | --- | --- | --- | --- | --- | --- | --- | --- | --- |
|  | Beta-lactam | Imipenem | 2.0% |  |  |  |  |  |  |  | 50.3% |  |  |  | 26.1% | 26.1% |
|  |  | Ceftiofur | 4.0% |  | 1.4% | 2.5% | 1.8% |  |  | 9.5% |  |  |  |  | 3.8% | 3.8% |
|  |  | Penicillin |  |  |  |  |  |  |  |  |  |  | 100.0% |  | 100.0% | 100.0% |
|  |  | Amoxycillin | 14.0% |  |  | 44.9% |  | 100.0% |  | 60.0% |  | 45.4% |  |  | 52.9% | 52.9% |
|  |  | Ampicillin | 14.0% | 89.5% |  |  | 11.5% |  | 68.7% | 69.5% |  |  | 0.0% |  | 42.2% | 42.2% |
|  |  | Piperacillin | 14.0% |  |  |  |  |  |  |  |  |  |  |  | 14.0% | 14.0% |
|  |  | Ceftazidime |  | 15.7% |  | 2.5% |  |  |  |  |  |  | 0.0% |  | 6.1% | 6.1% |
|  |  | Ceftriaxone |  |  |  |  |  |  |  |  | 9.8% |  |  |  | 9.8% | 9.8% |
|  |  | Cefoxitin |  |  |  | 0.8% |  |  |  |  |  |  |  |  | 0.8% | 0.8% |
|  |  | Cefazolin |  |  |  |  |  |  |  | 62.9% |  |  |  |  | 62.9% | 62.9% |
|  |  | Cephalexin |  | 5.3% |  |  |  |  |  |  |  | 10.8% |  |  | 8.1% | 8.1% |
|  |  | Cefepime |  |  |  | 2.5% |  |  |  |  |  |  |  |  | 2.5% | 2.5% |
|  |  | Cefotaxime |  |  |  | 2.5% |  |  |  |  |  |  |  | 23.2% | 12.9% | 12.9% |
|  |  | Meropenem |  |  |  |  |  |  |  |  | 82.7% |  |  |  | 82.7% | 82.7% |
|  |  | Aztreonam |  |  |  | 2.5% |  |  |  |  |  |  |  |  | 2.5% | 2.5% |
|  | Aminoglycosides | Gentamicin | 0.0% | 36.8% | 4.3% | 19.5% |  | 71.4% |  | 29.5% | 79.8% |  | 57.9% | 1.1% | 33.4% | 33.4% |
|  |  | Amikacin |  | 36.8% |  |  |  |  |  | 24.8% | 90.0% |  |  |  | 50.5% | 50.5% |
|  |  | Kanamycin |  | 31.6% |  |  |  |  |  | 32.4% |  |  | 21.1% |  | 28.4% | 28.4% |
|  |  | Neomycin |  | 31.6% |  |  |  |  |  |  |  | 24.7% | 0.0% |  | 18.8% | 18.8% |
|  |  | Streptomycin | 28.0% |  | 0.0% |  | 17.7% | 100.0% | 25.0% | 36.2% |  | 35.1% | 89.5% |  | 41.4% | 41.4% |
|  | Fluoroquinolones | Norfloxacin |  |  |  |  |  |  |  | 27.6% |  |  | 0.0% |  | 13.8% | 13.8% |
|  |  | Ciprofloxacin | 4.0% | 5.3% | 5.7% |  |  | 0.0% |  | 28.6% | 100.0% |  | 0.0% |  | 20.5% | 20.5% |
|  |  | Nalidixic acid | 2.0% |  | 7.1% | 13.6% |  |  |  |  |  |  |  |  | 7.6% | 7.6% |
|  |  | Enrofloxacin | 2.0% |  |  | 11.0% |  | 0.0% |  | 27.6% |  |  |  | 30.6% | 14.2% | 14.2% |
|  | Tetracyclines | Oxytetracycline |  |  |  |  |  |  |  | 71.4% |  |  |  |  | 71.4% | 71.4% |
|  |  | Tetracycline | 32.0% | 89.5% | 18.6% | 46.6% | 15.9% |  |  | 76.2% |  | 2.6% | 0.0% |  | 35.2% | 35.2% |
|  |  | Doxycycline |  |  |  |  |  |  |  | 47.6% |  |  | 0.0% |  | 23.8% | 23.8% |
|  | Macrolides | Azithromycin |  | 10.5% |  |  |  |  |  |  | 1.2% |  |  |  | 5.8% | 5.8% |
|  |  | Erythromycin |  |  |  |  |  |  |  |  |  |  | 89.5% |  | 89.5% | 89.5% |
|  | Lincosamide | Lincomycin | 100.0% |  |  |  |  |  |  |  |  |  |  |  | 100.0% | 100.0% |
|  | Sulfonamides | Sulfamethoxazole/Trimethoprim | 10.0% |  | 12.9% | 17.8% |  | 100.0% | 50.0% |  | 60.7% |  |  |  | 41.9% | 41.9% |
|  |  | Trimethoprim | 10.0% |  |  |  |  |  |  | 100.0% |  |  |  |  | 55.0% | 55.0% |
|  |  | Sulfamethoxazole | 100.0% |  |  |  |  |  |  |  |  |  |  |  | 100.0% | 100.0% |
|  |  | Sulphamethoxydiazine |  |  |  |  |  |  |  | 100.0% |  |  |  |  | 100.0% | 100.0% |
|  |  | Sulfadiazine |  |  |  |  |  |  |  | 99.0% |  |  |  |  | 99.0% | 99.0% |
|  | Amide alcohol | Florfenicol | 4.0% |  |  |  |  |  |  | 45.7% |  |  |  |  | 24.9% | 24.9% |
|  |  | Chloramphenicol |  |  | 17.1% | 11.0% |  |  |  | 41.0% | 50.3% |  |  |  | 29.8% | 29.8% |
|  | Polymyxins | Colistin sulfate | 14.0% |  |  | 0.0% |  |  |  | 25.7% | 9.8% |  |  |  | 12.4% | 12.4% |
|  |  | Polymyxin B | 32.0% |  |  |  |  |  |  |  |  |  |  |  | 32.0% | 32.0% |
|  | Sample size | | 50 | 19 | 70 | 118 | 113 | 14 | 16 | 105 | 173 | 77 | 19 | 353 |  |  |
|  | Countrys | | Vietnam | Bangladesh | Iran | Tunisia | Canada | Jordan | Ethiopia | China | Bangladesh | Slovakia | China | Turkiye |  |  |
|  | References | | My et al., 2023 [201] | Bag et al., 2021 [202] | Marashifard et al., 2019 [102] | Saidani et al., 2018 [203] | Majumder et al., 2021 [204] | Ismail et al., 2020 [103] | Messele et al., 2019 [205] | Zhao et al., 2024 [206] | Emon et al., 2024 [182] | Holko et al., 2019 [183] | Wang et al., 2024 [184] | Guner et al., 2024 [185] |  |  |

| *Streptococcus uberis* | **Antibiotics** | | **Resistance rate** | | | | | |  |  |  |  | **Average resistance rate** |
| --- | --- | --- | --- | --- | --- | --- | --- | --- | --- | --- | --- | --- | --- |
|  | Beta-lactam | Ampicillin | 9.2% |  |  | 89.9% | 1.0% | 28.6% |  | 28.6% |  |  | 31.5% |
|  |  | Amoxicillin/Clavulanic acid | 0.8% |  |  |  |  |  |  |  |  |  | 0.8% |
|  |  | Penicillin | 80.7% | 15.5% | 0.0% | 79.7% | 13.0% | 77.1% |  | 14.3% | 0.0% |  | 35.0% |
|  |  | Cloxacillin |  |  |  | 100.0% |  |  |  |  |  |  | 100.0% |
|  |  | Phenoxymethylpenicillin | 80.7% | 4.2% |  |  |  |  |  |  |  |  | 42.5% |
|  |  | Cephalotin | 0.8% |  |  |  |  |  |  |  |  |  | 0.8% |
|  |  | Ceftiofur | 3.4% | 2.8% | 19.3% |  | 2.0% | 1.5% |  | 14.3% |  |  | 7.2% |
|  |  | Ceftriaxone |  |  |  | 100.0% |  |  |  |  |  |  | 100.0% |
|  |  | Cephalexin |  |  |  | 100.0% |  |  |  |  |  |  | 100.0% |
|  |  | Cefoxitin |  |  |  |  |  |  | 18.0% |  |  |  | 18.0% |
|  |  | Cefazolin |  | 1.4% |  |  |  |  |  |  |  |  | 1.4% |
|  |  | Cefquinome |  | 1.4% |  |  |  |  |  |  |  |  | 1.4% |
|  |  | Cefoperazone |  | 2.8% |  | 26.0% |  |  |  |  |  |  | 14.4% |
|  | Aminoglycosides | Gentamicin | 1.7% |  | 0.0% | 20.3% |  |  |  |  | 96.0% |  | 29.5% |
|  |  | Kanamycin |  |  |  | 30.4% |  |  |  |  | 83.0% |  | 56.7% |
|  |  | Streptomycin |  |  |  | 87.0% |  |  | 98.4% |  |  |  | 92.7% |
|  |  | Spectinomycin |  |  |  |  |  |  | 3.3% |  |  |  | 3.3% |
|  |  | Novobiocin |  |  |  | 100.0% |  |  |  |  |  |  | 100.0% |
|  | Fluoroquinolones | Enrofloxacin | 9.2% | 8.5% |  | 21.7% |  |  |  | 50.0% | 0.0% |  | 17.9% |
|  |  | Ciprofloxacin |  |  |  |  |  |  | 0.0% |  |  |  | 0.0% |
|  | Tetracyclines | Tetracycline | 37.8% | 85.9% | 82.0% |  | 1.0% | 40.0% | 21.3% |  | 34.0% | 81.3% | 47.9% |
|  |  | Oxytetracycline |  |  |  |  |  |  |  | 92.9% |  |  | 92.9% |
|  | Macrolides | Erythromycin | 4.2% | 8.5% | 8.3% | 73.9% | 9.0% | 14.3% | 6.6% | 71.4% | 6.0% |  | 22.5% |
|  |  | Lincomycin |  | 93.0% |  |  |  |  |  |  |  |  | 93.0% |
|  |  | Clindamycin |  |  |  | 100.0% |  |  |  |  |  | 62.5% | 81.3% |
|  |  | Tiamulin |  |  |  |  |  |  | 3.3% |  |  |  | 3.3% |
|  | Lincosamide | Pirlimycin | 14.3% |  |  |  | 2.0% |  |  |  |  |  | 8.2% |
|  | Sulfonamides | Sulfamethoxazole |  | 18.3% |  |  |  |  | 100.0% |  |  |  | 59.2% |
|  | Chloramphenicols | Chloramphenicol |  | 33.3% |  |  |  |  | 0.0% |  |  |  | 16.7% |
|  | Other | Penicillin/Vancomycin | 1.7% |  |  |  |  | 1.4% |  |  |  |  | 1.6% |
|  | Sample size | | 119 | 71 | 228 | 69 | 215 | 70 | 61 | 14 | 53 | 16 |  |
|  | Countrys | | Brazil | Italy | Thailand | Egypt | Australia | Canada | Danish | Lebanon | Poland | China |  |
|  | References | | Fidelis et al., 2024 [210] | Monistero et al., 2021 [211] | Zhang et al., 2021 [212] | Abd El-Aziz et al., 2021 [122] | Langhorne et al., 2024 [213] | Cameron et al., 2016 [214] | Chehabi et al., 2019 [215] | Abboud et al., 2021 [216] | Kaczorek et al., 2017 [217] | Zhang et al., 2020 [123] |  |

| *Klebsiella pneumoniae* | **Antibiotics** | | **Resistance rate** | | | | | | **Average resistance rate** |
| --- | --- | --- | --- | --- | --- | --- | --- | --- | --- |
|  | Beta-lactam | Meropenem | 100.0% |  |  | 3.8% |  |  | 51.9% |
|  |  | Cefquinome |  |  |  |  | 4.8% |  | 4.8% |
|  |  | Imipenem | 20.6% |  |  |  |  |  | 20.6% |
|  |  | Cefuroxime | 33.3% | 4.6% |  |  |  |  | 18.9% |
|  |  | Amoxicillin-clavulanic acid |  |  |  |  | 7.1% |  | 7.1% |
|  |  | Cefoperazone |  | 1.5% |  |  |  |  | 1.5% |
|  |  | Cephalothin |  | 4.6% |  |  |  |  | 4.6% |
|  |  | Piperacillin |  | 3.0% |  |  |  |  | 3.0% |
|  |  | Cceftriaxone | 63.8% |  |  |  |  |  | 63.8% |
|  |  | Ampicillin | 11.3% |  |  |  |  |  | 11.3% |
|  |  | Ceftazidime |  | 1.5% |  |  |  |  | 1.5% |
|  |  | Cephalexin |  |  |  |  | 4.8% |  | 4.8% |
|  |  | Cefotaxime | 33.3% | 3.0% |  |  | 4.8% |  | 13.7% |
|  |  | Cefoxitin |  |  |  | 3.8% |  |  | 3.8% |
|  |  | Cefazolin |  |  |  | 31.3% |  |  | 31.3% |
|  | Aminoglycosides | Gentamicin | 77.3% | 12.1% | 4.2% |  |  | 5.9% | 24.9% |
|  |  | Streptomycin |  |  | 29.4% |  | 26.2% | 41.2% | 32.3% |
|  |  | Amikacin | 78.0% |  |  |  |  |  | 78.0% |
|  | Fluoroquinolones | Nalidixic acid | 53.9% |  |  |  |  |  | 53.9% |
|  |  | Ciprofloxacin | 75.2% |  |  |  |  |  | 75.2% |
|  | Tetracyclines | Tetracycline |  | 21.2% | 5.6% | 39.7% | 19.0% | 17.6% | 20.6% |
|  | Macrolides | Azithromycin | 34.0% |  |  |  |  |  | 34.0% |
|  | Sulfonamides | Sulfamethoxazole/Trimethoprim | 88.7% |  |  |  |  |  | 88.7% |
|  | Amide alcohol | Chloramphenicol | 34.8% | 13.6% |  |  |  |  | 24.2% |
|  | Polymyxins | Colistin sulfate | 7.8% |  |  |  |  |  | 7.8% |
|  | Sample size | | 141 | 66 | 143 | 131 | 42 | 68 |  |
|  | Countrys | | Bangladesh | China | United States | China | Scottish | China |  |
|  | References | | Emon et al., 2024 [182] | Yang et al., 2021 [207] | Yang et al., 2019 [150] | Cai et al., 2025 [208] | Pollock et al., 2025 [209] | Xu et al., 2022 [142] |  |

| *Streptococcus dysgalactiae* | **Antibiotics** | | **Resistance rate** | | | | | | **Average resistance rate** |
| --- | --- | --- | --- | --- | --- | --- | --- | --- | --- |
|  | Beta-lactam | Penicillin |  |  |  | 100.0% | 0.0% | 12.7% | 37.6% |
|  |  | Ampicillin |  |  |  | 14.8% |  |  | 14.8% |
|  |  | Ceftriaxone | 13.6% |  |  |  |  |  | 13.6% |
|  |  | Ceftazidime |  |  |  | 0.0% |  |  | 0.0% |
|  |  | Cephalexin | 34.1% |  |  |  |  |  | 34.1% |
|  |  | Cefotaxime |  | 45.0% |  |  |  |  | 45.0% |
|  | Aminoglycosides | Streptomycin | 58.0% |  | 27.9% | 100.0% |  |  | 62.0% |
|  |  | Kanamycin | 89.8% |  |  | 0.0% | 51.0% |  | 46.9% |
|  |  | Neomycin |  |  | 52.4% | 0.0% |  |  | 26.2% |
|  |  | Gentamicin |  |  |  | 77.8% | 68.0% | 11.3% | 52.4% |
|  | Fluoroquinolones | Enrofloxacin |  |  | 28.0% |  | 5.0% | 14.1% | 15.7% |
|  |  | Ciprofloxacin |  |  |  | 0.0% |  |  | 0.0% |
|  |  | Norfloxacin |  |  |  | 0.0% |  |  | 0.0% |
|  | Tetracyclines | Tetracycline |  | 100.0% | 100.0% | 100.0% | 61.0% |  | 90.3% |
|  |  | Doxycycline |  |  |  | 0.0% |  |  | 0.0% |
|  |  | Oxytetracycline |  |  | 89.9% |  |  |  | 89.9% |
|  | Macrolides | Erythromycin |  | 36.7% |  | 100.0% | 22.0% |  | 52.9% |
|  | Sulfonamides | Sulfamethoxazole |  | 18.3% |  |  |  |  | 18.3% |
|  | Amide alcohol | Chloramphenicol |  | 33.3% |  |  |  |  | 33.3% |
|  | Sample size | | 88 | 60 | 89 | 27 | 41 | 71 |  |
|  | Country | | China | China | New Zealand、USA | China | Poland | Turkiye |  |
|  | References | | Zhang et al., 2018 [218] | Shen et al., 2021 [152] | Petrovski et al., 2015 [219] | Wang et al., 2024 [184] | Kaczorek et al., 2017 [198] | Guner et al., 2024 [185] |  |
